# Activation of Gcn2 by small molecules designed to be inhibitors

**DOI:** 10.1016/j.jbc.2023.104595

**Published:** 2023-03-08

**Authors:** Kenneth R. Carlson, Millie M. Georgiadis, Feven Tameire, Kirk A. Staschke, Ronald C. Wek

**Affiliations:** 1 Department of Biochemistry and Molecular Biology, Indiana University School of Medicine, Indianapolis, Indiana, USA; 2 Indiana University Melvin and Bren Simon Comprehensive Cancer Center, Indianapolis, Indiana, USA; 3 HiberCell, Inc, New York, New York, USA

**Keywords:** integrated stress response, Gcn2, eIF2 kinase, eIF2 phosphorylation

## Abstract

The integrated stress response (ISR) is an important mechanism by which cells confer protection against environmental stresses. Central to the ISR is a collection of related protein kinases that monitor stress conditions, such as Gcn2 (EIF2AK4) that recognizes nutrient limitations, inducing phosphorylation of eukaryotic translation initiation factor 2 (eIF2). Gcn2 phosphorylation of eIF2 lowers bulk protein synthesis, conserving energy and nutrients, coincident with preferential translation of stress-adaptive gene transcripts, such as that encoding the Atf4 transcriptional regulator. While Gcn2 is central for cell protection to nutrient stress and its depletion in humans leads to pulmonary disorders, Gcn2 can also contribute to the progression of cancers and facilitate neurological disorders during chronic stress. Consequently, specific ATP-competitive inhibitors of Gcn2 protein kinase have been developed. In this study, we report that one such Gcn2 inhibitor, Gcn2iB, can activate Gcn2, and we probe the mechanism by which this activation occurs. Low concentrations of Gcn2iB increase Gcn2 phosphorylation of eIF2 and enhance *Atf4* expression and activity. Of importance, Gcn2iB can activate Gcn2 mutants devoid of functional regulatory domains or with certain kinase domain substitutions derived from Gcn2-deficient human patients. Other ATP-competitive inhibitors can also activate Gcn2, although there are differences in their mechanisms of activation. These results provide a cautionary note about the pharmacodynamics of eIF2 kinase inhibitors in therapeutic applications. Compounds designed to be kinase inhibitors that instead directly activate Gcn2, even loss of function variants, may provide tools to alleviate deficiencies in Gcn2 and other regulators of the ISR.

The Integrated Stress Response (ISR) is critical for cell protection against environmental insults. A primary feature of the ISR is phosphorylation of the α subunit of eukaryotic initiation factor 2 (eIF2), which rapidly reduces protein synthesis, allowing cells to maximize resources towards reprograming of gene expression that is designed to alleviate stress damage and restore cell homeostasis (1). While induced phosphorylation of eIF2α (p-eIF2α) can lower bulk translation, certain mRNAs, such as those encoding the transcription regulator Atf4, are preferentially translated to foster expression of ISR-target genes. In this way, the ISR confers both translational and transcriptional control directed at precise levels and times in response to stress.

A family of eIF2α kinases serves as first responders in the ISR, each being activated by distinct cell stresses. To monitor stress, the eIF2α kinases have different regulatory regions that associate with ligands or interactive proteins whose levels sharply change during stress, inducing ordered mechanisms of activation that include dimerization, trans interdimer phosphorylation at the kinase activation loop, and eventual eIF2α substrate recognition and phosphorylation. For example, the eIF2α kinase Gcn2 (EIF2AK4) is activated by starvation for amino acids and stresses that trigger ribosome stalling, including UV irradiation and certain oxidative stresses (1, 2, 3, 4, 5, 6). For recognition of these diverse stresses, Gcn2 has an amino-terminal RWD-protein interaction domain, pseudo kinase and protein kinase domains, a region related to the histidyl tRNA synthetase (HARS), and a carboxy-terminal domain (CTD) that forms an interdigitated dimer and is suggested to facilitate Gcn2 binding to ribosomes (7, 8, 9) (Fig. 1*A*). Uncharged tRNAs that accumulate during amino acid depletion are suggested to bind to the HARS-related region of Gcn2, facilitating activation of the adjacent kinase domain for induced p-eIF2α (10, 11). Gcn2 association with ribosomes is suggested to be critical for its recognition and binding of uncharged tRNAs, and this process involves the accessory protein Gcn1, which engages with the RWD of this eIF2α kinase (9, 12, 13, 14). Furthermore, Gcn1 is suggested to contribute to activation of Gcn2 by association with translating ribosomes that stall and collide during certain stresses (4, 5, 6, 9, 15, 16).

**Figure 1:**
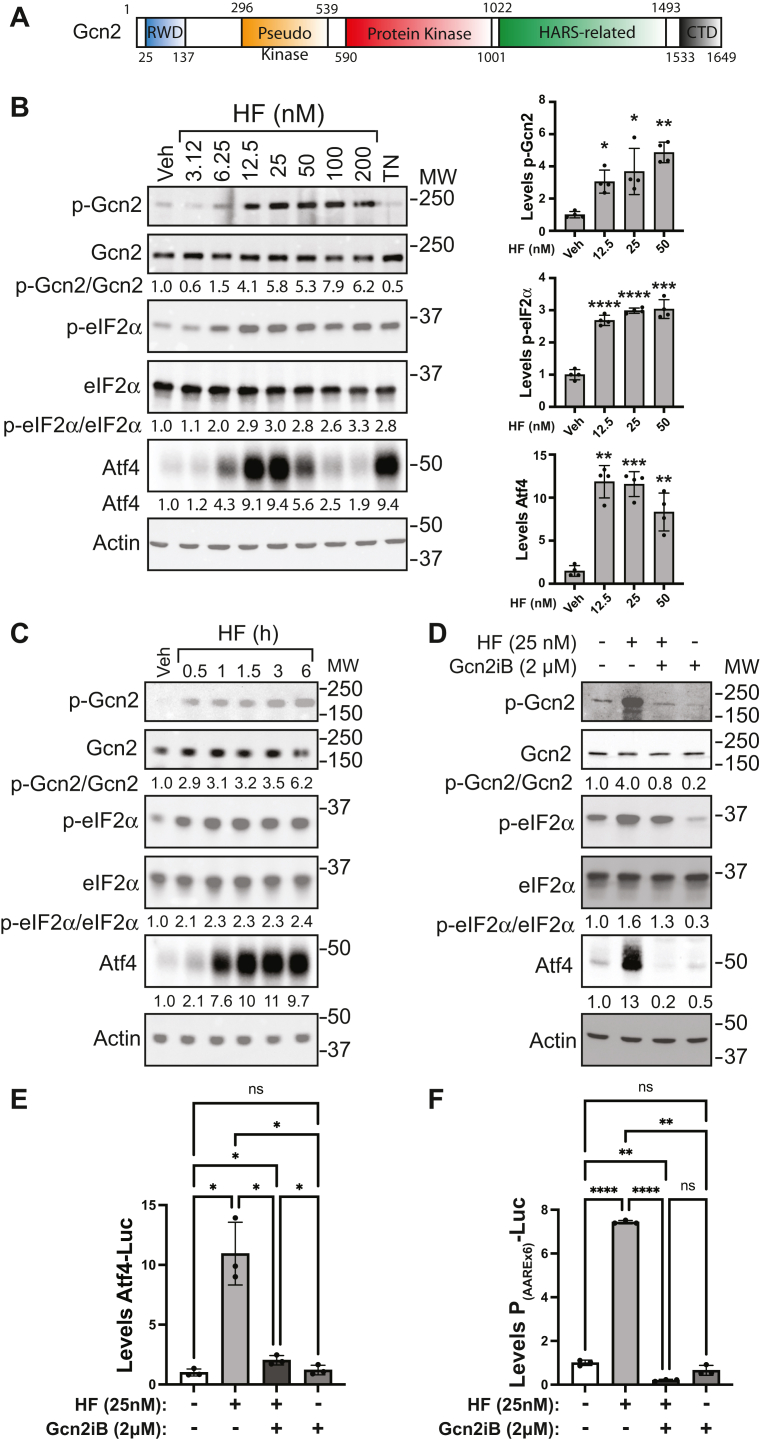
**HF induces the ISR and this induction can be blocked by Gcn2iB.***A*, illustration of the domains of Gcn2, with the residues indicated for each domain in the human Gcn2. *B*, HEK293 cells were treated with the indicated concentrations of halofuginone (HF), 2 μM tunicamycin (TN), or vehicle (Veh) for 6 h, and the levels of total and phosphorylated Gcn2 (T899), total and phosphorylated of eIF2α (S51), Atf4, and actin were measured by immunoblot analyses. Relative levels of these proteins measured in four immunoblot experiments are represented in the bar graphs. Statistical analyses were performed using one-way Welch’s ANOVA with follow up tests comparing each treatment to Veh-treated cells and using Benjamini-Hochberg FDR correction for multiple comparisons. Statistical significance is indicated by ∗ symbols with ∗ = q < 0.05, ∗∗ = q < 0.01, ∗∗∗ = q < 0.001, ∗∗∗∗ = q < 0.0001. *C*, HEK293 cells were treated with 25 nM HF for up to 6 h, as indicated, and the levels of the indicated total and phosphorylated proteins were measured by immunoblot. *D*, HEK293 cells were treated with vehicle (Veh), 25 nM HF alone, 25 nM HF + 2 μM Gcn2iB, or 2 μM Gcn2iB alone for 6 h, and lysates were collected and the indicated phosphorylated and total proteins were measured by immunoblot analyses. For panels *A*–*C*: quantitation was performed by densitometry using ImageJ software, and the values for quantitation appear below each of the respective blots. Phosphorylated Gcn2 and eIF2α were first normalized to total Gcn2 and eIF2α, then p-Gcn2/Gcn2, p-eIF2α/eIF2α, and Atf4 were normalized to the Veh-treated (leftmost) lane. *E*, HEK293 cells were transfected with Atf4-Luc plasmids and nano-luciferase for normalization, and cells were treated with 25 nM HF alone, 25 nM HF + 2 μM Gcn2iB, or 2 μM Gcn2iB alone for 6 h. Atf4-Luc activities were determined and are presented in a bar graph normalized to vehicle (−/−). Error bars represent the SD of n = 3 biological replicates. *F*, HEK293 cells with stably integrated P_(AAREx6)_-Luciferase reporter were treated with 25 nM HF alone, 25 nM HF + 2 μM Gcn2iB, or 2 μM Gcn2iB alone, and P_(AAREx6)_-Luc was measured after 6 h and presented in a bar graph normalized to vehicle (−/−). Error bars represent the SD of n = 3 biological replicates. For panels E and F: ∗ indicates a statistically significant change in luciferase activity as determined by one-way Welch’s ANOVA with follow up tests comparing each treatment to each other treatment and using Benjamini-Hochberg FDR correction for multiple hypothesis testing where ∗ = q < 0.05, ∗∗ = q < 0.01, ∗∗∗∗ = q < 0.0001. The ns indicates not significant. ISR, integrated stress response.

Emphasizing the importance of Gcn2 in stress adaptation, mice deleted for Gcn2 have increased morbidity when fed a diet depleted of amino acids (17). Human patients with loss of function Gcn2 mutations have pulmonary disorders, including pulmonary veno-occlusive disease (PVOD), pulmonary arterial hypertension, and pulmonary capillary hemangiomatosis (18, 19, 20, 21). Reported disease causing *Gcn2* mutations are nonsense mutations or frameshifts that introduce premature stop codons, along with missense mutations (21). The basis for why Gcn2 deficiency leads to pulmonary disorders in humans is not yet clear, but lungs can be challenged by a range of stress agents, including inhaled toxicants and microbes, and Gcn2 and the ISR is suggested to provide for cell resistance to these insults and remodeling of the pulmonary vasculature. Therefore, restoring Gcn2 function in these patient populations would have therapeutic utility.

While loss of Gcn2 function can contribute to disease, chronic activation of Gcn2 is also maladaptive. For example, certain mutations in aminoacyl tRNA synthetase genes cause peripheral neuropathies and Charcot-Marie-Tooth (CMT) disease (5, 22, 23). In this case, the underlying synthetase mutations result in deficiencies in tRNA charging that lead to constitutive activation of Gcn2. The chronic induction of p-eIF2α is suggested to be a contributor to these pathologies, and targeted loss of Gcn2 activity can alleviate the progression of CMT. Inhibition of Gcn2 is also a therapeutic strategy for treatment of certain cancers. Androgen-sensitive and castration-resistant prostate cancers require Gcn2 to maintain amino acid transporter gene expression for essential amino acids to support tumor growth (24). Furthermore, genetic or pharmacological loss of Gcn2 was reported to reduce proliferation of prostate cancer cells in cell culture and mouse models of tumor growth (24).

Studies focused on strategies to develop small molecule inhibitors of Gcn2 have resulted in the identification of compounds that target the nucleotide-binding cleft, which preclude ATP association (25, 26, 27). By convention, these inhibitor-binding modes have been categorized as types I, II, III, IV depending on whether the small molecule binds competitively with ATP using the ‘DFG-in’ (type I) conformation or the ‘DFG-out’ (type II) conformation or noncompetitively by distal engagement with the ATP-binding pocket (types III and IV) (28). Gcn2iB is an ATP-competitive inhibitor of Gcn2 that was described for use in combination with asparaginase treatment, a potent trigger of asparagine depletion, for treatment of lymphoid and myeloid leukemias and pancreatic cancer (26). The crystal structure of Gcn2 in complex with Gcn2iB reveals a type I ½ binding mode which features a DFG-in, αC helix-out conformation (27). Of importance, several receptor tyrosine kinase ATP-competitive inhibitors (RTKi) with type I ½–binding modes have been reported to induce Gcn2 activity, and it is proposed that this stimulation occurs through inhibitor binding to one protomer of a kinase domain dimer inducing a conformation that stimulates the kinase activity of the adjacent protomer (29). The phenomenon of an ATP-competitive inhibitor–stimulating enzyme activity through binding to the ATP-binding site, termed “paradoxical activation”, has also been observed for the kinase B-RAF (30).

In this study, we addressed the nature of the small molecule activation of Gcn2 and whether specific pharmacological applications of the inhibitor Gcn2iB can instead function as an activator of Gcn2. We show that low concentrations of Gcn2iB are potent inducers of Gcn2 and p-eIF2α independent of stress. While the Gcn2 regulatory domains are essential for activation in response to stress involving elevated levels of uncharged tRNAs, the regions are dispensable for activation by Gcn2iB. Finally, we show that Gcn2iB activation can restore induction of the ISR in cells expressing Gcn2 mutant proteins described for a patient with a PVOD lung disorder.

## Results

Halofuginone (HF) potently activates Gcn2 by inhibiting aminoacylation of tRNA^Pro^ (31, 32). To establish the dose-response relationship of HF for activation of Gcn2 in HEK293 cells, we treated these cells with up to 200 nM HF for 6 h (Fig. 1*B*). Upon treatment with HF, there was an increased activation of Gcn2, as measured by autophosphorylation (T899), and p-eIF2α (S51) in immunoblot analyses (Fig. 1*B*). Furthermore, there was enhanced expression of the ISR effector Atf4, whose synthesis is increased upon p-eIF2α (33). In contrast to p-Gcn2 and p-eIF2α, whose levels continued to be enhanced with increasing HF concentration, increased amounts of Atf4 protein reached a peak between 12.5 nM and 25 nM of HF. At 100 nM of HF, there was a significant reduction in Atf4 levels, which is consistent with the idea that high levels of HF can lower charging of tRNA^Pro^ to levels that are insufficient for preferential translation in ISR genes (32). As a control, we also induced the ISR by treatment with tunicamycin (TN), a potent endoplasmic reticulum stress agent that activates an alternative eIF2α kinase PERK. As expected, there was induced p-eIF2α and Atf4, but minimal phosphorylation of Gcn2 (Fig. 1*B*), further supporting the specificity of HF as a potent activator of Gcn2.

We next addressed the timing of induced Gcn2 and the ISR. HEK293 cells were treated with the 25 nM of HF and levels of p-Gcn2, p-eIF2α, and Atf4 were measured for up to 6 h (Fig. 1*C*). There was increased p-Gcn2 and p-eIF2α within 30 min of the treatment, which was sustained for up to 6 h. Expression of Atf4 was enhanced at 1 h of HF exposure, with further accumulation after 1.5 h of treatment. These results are consistent with accumulating uncharged tRNA rapidly inducing Gcn2 phosphorylation of eIF2α, followed thereafter by preferential translation of preexisting *Atf4* mRNA.

Given that Gcn2iB is reported to be an ATP-competitive inhibitor of Gcn2, we determined whether the induction of the ISR observed upon HF treatment can be blocked by Gcn2iB. HEK293 cells were treated with 25 nM HF for 6 h in the presence or absence of 2 μM Gcn2iB, a concentration reported to be effective for the inhibition of Gcn2 (24). There were increased amounts of p-Gcn2, p-eIF2α, and Atf4 in cells treated with HF alone but not in cells treated with both HF and Gcn2iB (Fig. 1*D*).

We also addressed the inhibitory function of Gcn2iB using *Atf4* translational expression and activity reporters. The Atf4-Luc reporter consists of the encoded 5′-leader of the *Atf4* mRNA inserted between the constitutive CMV promoter and the firefly luciferase-coding sequence and is preferentially translated upon p-eIF2α (33). There was robust induction of Atf4-Luc expression in cells treated with HF, which was sharply diminished when Gcn2iB was applied in combination (Fig. 1*E*). We also utilized HEK293 cells stably expressing an Atf4 activity reporter. This reporter-designated P_(AAREx6)_-Luc encodes six contiguous amino acid response elements (C/ebp/Atf4 consensus-binding elements) upstream of a luciferase reporter gene and is a measure of Atf4-directed transcription. The P_(AAREx6)_-Luc–expressing cells were treated with HF and similarly showed robust induction that was sharply reduced in the presence of Gcn2iB (Fig. 1*F*). Together, these results show that 2 μM Gcn2iB is a potent inhibitor of Gcn2 and the ISR in cells treated with HF.

### Gcn2iB can induce Gcn2 activity

**Figure 2:**
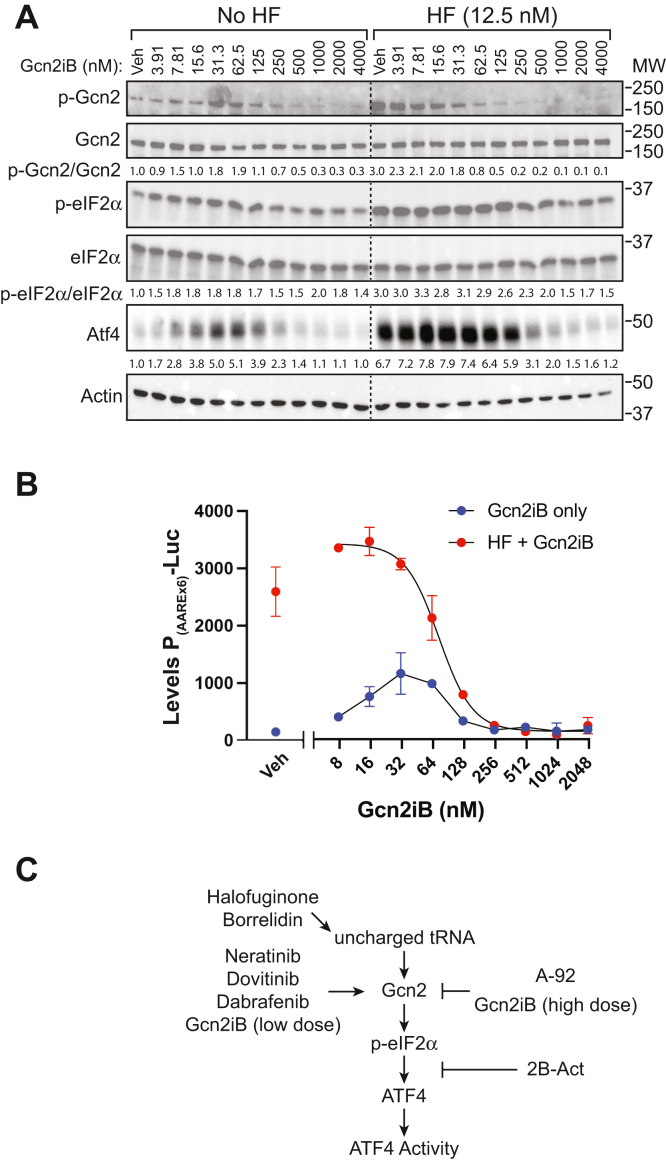
**Gcn2iB dose response for modulation of Gcn2 activity.***A*, HEK293 cells were pretreated with the indicated concentrations of Gcn2iB for 30 min and then treated with 12.5 nM HF or vehicle (No HF) for 6 h. Protein lysates were collected and the amounts of p-Gcn2 (T899), total Gcn2, p-eIF2α (S51), total eIF2α, Atf4, and actin were measured by immunoblot. Protein levels were quantitated by densitometry and are presented below each lane of their respective blots. Phospho-Gcn2 and p-eIF2α were first normalized to their corresponding total protein blots and p-Gcn2/Gcn2, p-eIF2α/eIF2α, and Atf4 are presented relative to vehicle-treated samples. A representative example from n = 2 independent experiments is shown. *B*, HEK293 cells expressing P_(AAREx6)_-Luc were pretreated with the indicated concentrations of Gcn2iB for 30 min, followed by treatment with 12.5 nM HF or vehicle (Gcn2iB only) for 6 h. Lysates were collected and luciferase activity was measured. Error bars represent the SD of three biological samples. Result shown is a representative example of n = 2 independent experiments. *C*, schematic of Gcn2 and ISR regulation by small molecules. Halofuginone (HF) and borrelidin inhibit aminoacylation of tRNA^Pro^ and tRNA^Thr^, respectively, triggering increases in uncharged tRNAs that lead to activation of Gcn2. Low doses of Gcn2iB and other kinase inhibitors neratinib, dovitinib, and dabrafenib can activate Gcn2, leading to increased phosphorylation of p-eIF2α and enhanced expression of the downstream ISR effector, Atf4. By contrast, high doses of Gcn2iB and A-92 inhibit Gcn2 despite activation by uncharged tRNAs and lower p-eIF2α and Atf4 expression and activity. ISR, integrated stress response.

Gcn2 activation by ATP competitive kinase inhibitors has been reported previously using EGFR inhibitors (29), and we wished to determine whether such a mechanism can also be triggered by a small molecule inhibitor specifically designed to target Gcn2. To explore the concentration-dependent effects of Gcn2iB on ISR activation, HEK293 cells were treated with Gcn2iB from 4 nM to 4 μM in the presence or absence of HF treatment for 6 h (Fig. 2*A*). HF treatment resulted in increased amounts of p-Gcn2, p-eIF2α, and Atf4, each of which were diminished with higher concentrations of Gcn2iB. There were reductions in induced p-Gcn2 at 62.5 nM Gcn2iB or greater and Atf4 beginning at 250 nM Gcn2iB Figure 2*A*. Of importance, cells treated with Gcn2iB in the absence of HF exhibited a biphasic response for induction of p-Gcn2, p-eIF2α, and Atf4 with increases for all three ISR markers observed in the range of 10 to 100 nM Gcn2iB. Above 250 nM Gcn2iB, the levels of p-Gcn2 were lowered below that determined for vehicle, and Atf4 levels were also not appreciably induced. These results indicate that although Gcn2iB can act to inhibit Gcn2 stimulated by HF treatment, in the absence of an external stress, lower concentrations of Gcn2iB instead activate Gcn2 and the ISR.

To further test whether Gcn2iB can induce the ISR, P_(AAREx6)_-Luc–expressing cells were treated with increasing concentrations of Gcn2iB in the presence and absence of HF (Fig. 2*B*). Treatment with HF resulted in an approximately 30-fold increase in luciferase signal, which was diminished with increasing concentrations of Gcn2iB, with an IC_50_ of 73 nM Gcn2iB. When Gcn2iB was administered to cells in the absence of HF, there was a biphasic induction of luciferase activity with a peak induction of 7.8-fold at about 32 nM Gcn2iB. As will also be detailed fully in the later experiments that analyze Gcn2 structure/function, Gcn2iB also induced *Atf4* translation as judged by the Atf4-Luc translation reporter (Fig. S1). To ensure that Gcn2iB induction of *Atf4* translational expression is dependent on p-eIF2α, we cotransfected the Atf4-Luc reporter with a plasmid-encoding elevated levels of Gadd34, which targets protein phosphatase I dephosphorylation of p-eIF2α as part of the feedback regulation of the ISR (1, 24, 34). As anticipated, coexpression of Gadd34 significantly lowered Atf4-Luc expression in response to activating concentrations of Gcn2iB (Fig. S1). These results support the idea that Gcn2iB can activate Gcn2 as measured by ISR biomarkers and increased Atf4 translational expression and transcriptional activity in the absence of external stress (Fig. 2*C*). When Gcn2 activity is stimulated by increased levels of uncharged tRNA as triggered by HF treatment, Gcn2iB acts as a typical ATP-competitive inhibitor, lowering p-eIF2α and the expression and activity of its downstream effector, Atf4 (Fig. 2*C*).

### Induction of the ISR by Gcn2iB is blocked by pharmacological and genetic loss of Gcn2

**Figure 3:**
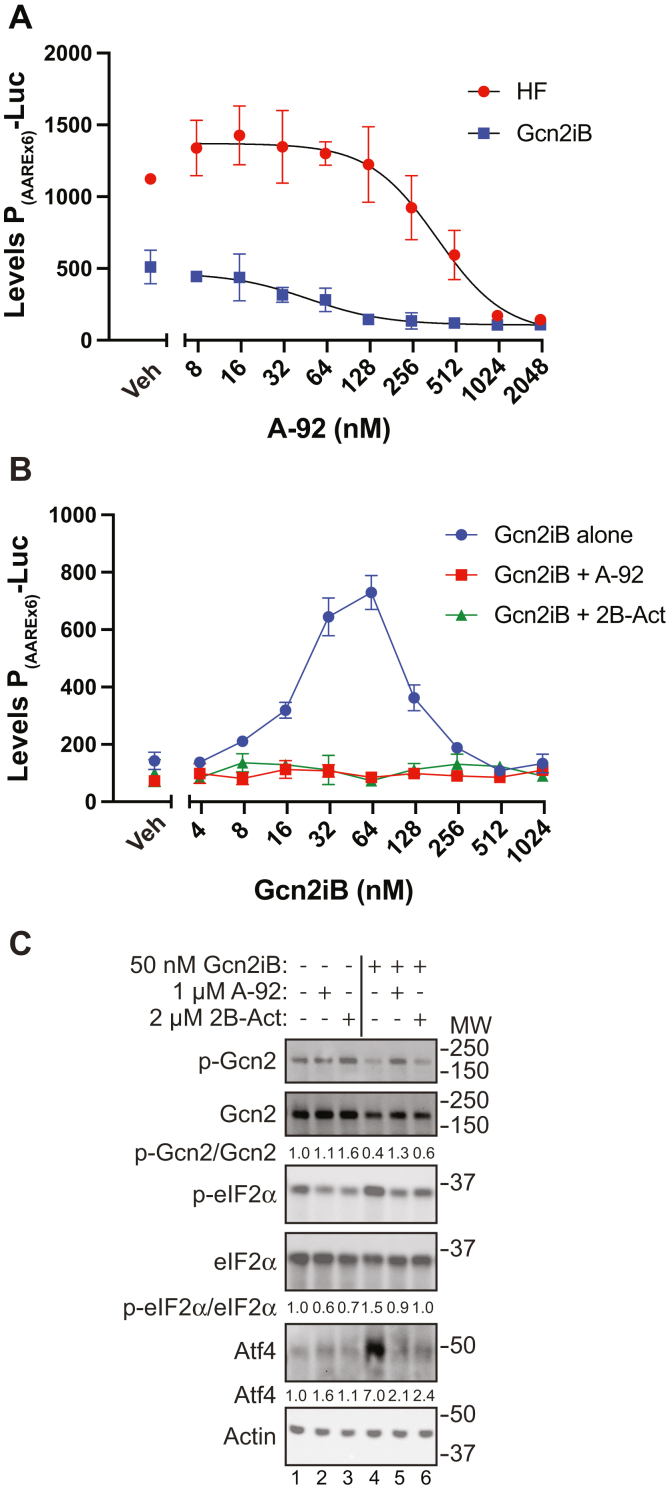
**Induction of the ISR by Gcn2iB requires Gcn2 phosphorylation of eIF2α.***A*, cells expressing P_(AAREx6)_-Luc were pretreated with the indicated concentrations of A-92 for 30 min and then 12.5 nM HF or 31.25 nM Gcn2iB were added for 6 h. Luciferase activity was measured. Error bars represent the SD of three biological replicates. *B*, cells expressing P_(AAREx6)_-Luc were pretreated with 1 μM A-92, 2 μM 2B-Act, or vehicle (Gcn2iB alone) for 30 min; then the indicated concentration of Gcn2iB was added and luciferase activity was measured after 6 h. Error bars represent the SD of three biological replicates. *C*, HEK293 cells were pretreated with 1 μM A-92, 2 μM 2B-Act, or vehicle for 30 min and then 50 nM Gcn2iB was added for 6 h. Protein lysates were prepared and the levels of total and p-Gcn2 (T899), total and p-eIF2α (S51), Atf4, and actin were measured by immunoblot analyses. Quantitation for p-Gcn2, p-eIF2α, and Atf4 are shown below each of the respective blots. Phosphorylated Gcn2 and eIF2α were first normalized to total Gcn2 or eIF2α of their respective samples and then normalized to Veh-treated (leftmost) sample. Results shown are representative of two independent experiments. ISR, integrated stress response; HF, Halofuginone.

To determine the contributions of Gcn2 eIF2α kinase activity in the induction of the ISR by HF and Gcn2iB, we treated HEK293 cells expressing P_(AAREx6)_-Luc with increasing concentrations of the type I ATP-competitive Gcn2 inhibitor A-92 after being activated by either 12.5 nM HF or 31.25 nM Gcn2iB (Fig. 3*A*). Treatment with A-92 at concentrations greater than 125 nM significantly reduced P_(AAREx6)_-Luc activity in cells induced by HF and Gcn2iB, indicating that Gcn2 activity was responsible for the increased Atf4 activity upon HF or Gcn2iB treatment. To address whether induced p-eIF2α confers the Gcn2iB induction of Atf4 activity, we used 2B-Act, a derivative of ISRIB and a small molecule activator of eIF2B (35, 36). The eIF2B catalyzes the exchange of eIF2-GDP to eIF2-GTP, and 2B-Act renders this guanine nucleotide exchange factor largely insensitive to p-eIF2α (36, 37, 38, 39). Cells expressing P_(AAREx6)_-Luc were treated with increasing concentrations of Gcn2iB either alone or in combination with 1 μM A-92 or 2 μM 2B-Act, and luciferase activity was measured (Fig. 3*B*). The biphasic induction of luciferase activity, which was observed when the cells were exposed to Gcn2iB alone, was ablated in the presence of either A-92 or 2B-Act (Fig. 3*B*). HEK293 cells were treated with A-92 or 2B-Act alone or in combination with Gcn2iB, and ISR markers were measured by immunoblot analyses (Fig. 3*C*). Treatment with A-92 or 2B-Act lowered p-eIF2α below basal levels in untreated cells but did not alter p-Gcn2 or Atf4 levels, which were minimal in the nonstressed conditions. As described above, treatment with Gcn2iB alone increased the levels of p-eIF2α and Atf4, but diminished p-Gcn2 (Fig. 3*C*, lane 4). Of importance, Gcn2iB cotreatment with either A-92 or 2B-Act lowered p-eIF2α and Atf4 levels (Fig. 3*C*, lanes five and 6). These results are consistent with the lowered P_(AAREx6)_-Luc activity in the cotreatment regimen and support the model that Gcn2iB induces p-eIF2α by Gcn2, leading to reduced eIF2B GEF activity and eIF2-GTP, which would trigger enhanced Atf4 protein synthesis and activity (Fig. 2*C*).

**Figure 4:**
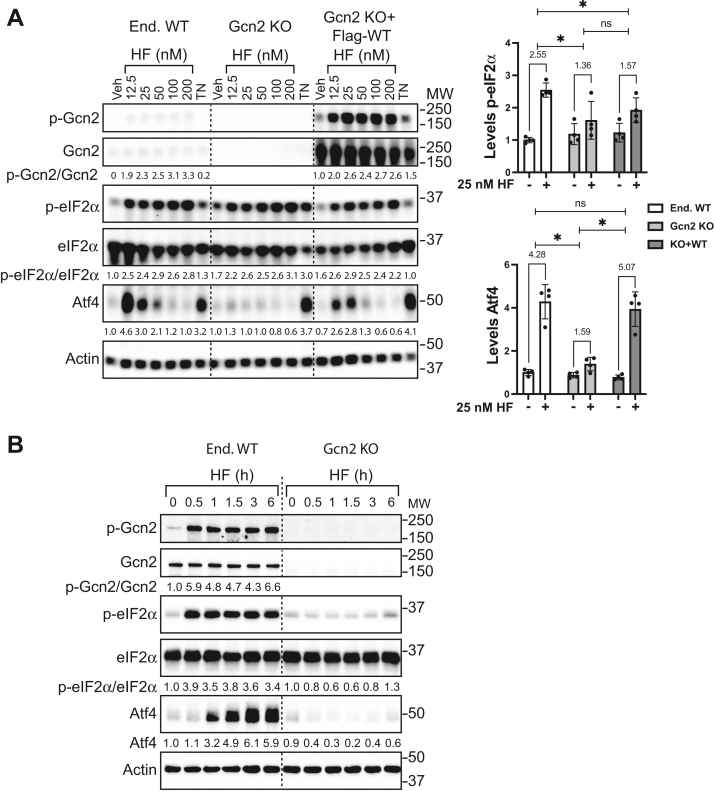

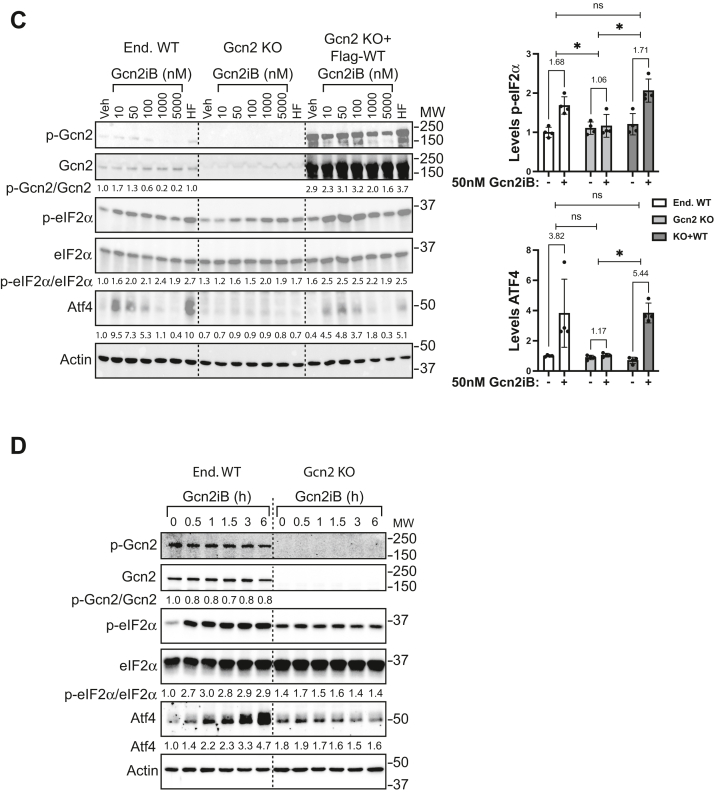
**Induction of the ISR by Gcn2iB requires Gcn2 protein expression.***A*, HEK293 cells containing WT Gcn2 (End. WT), Gcn2 KO, or Gcn2 KO transfected with WT Gcn2 expression plasmid were treated with the indicated concentrations of halofuginone (HF) or 2 μM tunicamycin (TN) for 6 h. Total and phosphorylated Gcn2 (T899), total and phosphorylated eIF2α (S51), Atf4, and actin were measured by immunoblot analyses. Phosphorylated Gcn2/total Gcn2 was normalized to Gcn2 KO+Flag-WT Veh, while p-eIF2α/total ratio and Atf4 were each normalized to End. WT Veh. Bar graphs displaying quantitation for p-eIF2α and Atf4 upon Veh and 25 nM HF treatment are shown to the *right* with values obtained from the displayed immunoblot along with three additional biological replicates with error bars representing the SD. The fold change upon treatment is displayed above each group with significance stars indicating *p* < 0.05 from Welch’s *t* test performed on the fold change. *B*, HEK cells with endogenous Gcn2 (End WT) and Gcn2 KO cells were treated with the mock transfection protocol, followed by treatment with 25 nM HF for the indicated times. The indicated phosphorylated and total proteins were then measured by immunoblot analyses. Levels of the phosphorylated proteins were first normalized to their respective total proteins and then to End WT Veh-treated cells. The results shown are representative of two independent experiments. *C*, HEK293T with WT Gcn2 (End. WT), Gcn2 KO, or Gcn2 KO transfected with WT Gcn2 expression plasmid were treated with the indicated concentrations of Gcn2iB or 25 nM HF for 6 h. The indicated proteins were then measured by immunoblot analysis. Levels of phosphorylated Gcn2 and eIF2α, along with total amounts of Atf4, were each normalized to End WT Veh-treated sample. Bar graphs displaying the levels of p-eIF2α and Atf4 upon Veh (−) or 50 nM Gcn2iB (+) treatment are shown to the right. Error bars represent the SD of n = 4 biological replicates, and fold changes upon treatment with 50 nM Gcn2iB are displayed above each grouping. The fold changes upon Gcn2iB treatment were compared using Welch’s *t* test with ∗ indicating *p* < 0.05. *D*, WT and Gcn2 KO HEK cells were treated with 50 nM Gcn2iB for the indicated times and the levels of the indicated total and phosphorylated proteins were measured by immunoblot analyses. Levels of p-Gcn2, p-eIF2α, and Atf4 were each normalized to WT 0 h sample. Results shown are representative of n = 2 independent experiments. ISR, integrated stress response.

To further test the idea that Gcn2iB directly activates Gcn2, we generated a knock-out of Gcn2 in HEK293 cells by CRISPR/Cas9 gene editing and isolated clonal populations of these cells. HEK293 cells expressing endogenous WT Gcn2 (End WT), Gcn2 KO, and Gcn2 KO cells rescued by transient expression of amino terminal flag-tagged Gcn2 were treated with increasing concentrations of HF for 6 h (Fig. 4*A*). As a control, this collection of cells were also treated with 2 μM TN, which causes endoplasmic reticulum stress and activation of another eIF2α kinase PERK. In cells bearing endogenous Gcn2, there was increased p-Gcn2, p-eIF2α, and Atf4 expression in response to HF treatment, whereas TN enhanced only p-eIF2α and Atf4 as expected by this stress, triggering another eIF2α kinase (Fig. 4*A*). As noted earlier, the Atf4 protein levels were only enhanced at lower concentrations of HF (Fig. 1*A*). In contrast, there was no increase in Atf4 expression in Gcn2 KO cells subjected to HF treatment, whereas TN treatment led to robust Atf4 expression (Fig. 4*A*). When Gcn2 KO cells were rescued with Flag-Gcn2 by transient transfection, p-Gcn2 and Atf4 were induced during HF treatment. These results are consistent with HF being a potent inducer of Gcn2 and illustrate a rescue model for Gcn2 structure/function analyses that will be exploited below.

It is important to note that there can be some increased p-eIF2α by HF treatment at later time points (6 h) independent of the functional status of Gcn2. The reason for this observation is twofold. First, deletion of a primary eIF2α kinase activated by a dedicated stress can trigger induction of secondary eIF2α kinase(s), albeit with a significant delay (40, 41). Second, the nature of the transfection protocol used in the rescue assays appears to cause some underlying stress that is contributing to some p-eIF2α. Together, these factors contribute to some underlying p-eIF2α that is visualized in a time course of HF treatment of End WT Gcn2 and Gcn2 KO cells (Fig. 4*B*). There was induced p-Gcn2 and Atf4 expression that is fully dependent on Gcn2 expression, but there were appreciable p-eIF2α levels during the course of HF treatment in the Gcn2 KO cells. As a consequence, measurements of *Atf4* translation, Atf4 protein levels, and Atf4 transcriptional activity rather than p-eIF2α were used in subsequent experiments to measure Gcn2 activation by HF.

We next analyzed the Gcn2 KO and its WT counterpart for activation by Gcn2iB. Treatment with Gcn2iB at low concentrations (10 and 50 nM) for 6 h increased p-eIF2α and Atf4 protein levels in cells containing endogenous Gcn2 and in Gcn2 KO cells transfected with WT Gcn2 plasmid but not in Gcn2 KO cells transfected with empty vector (Fig. 4*C*). Treatment with Gcn2iB for up to 6 h showed a 2.7-fold induction of p-eIF2α in WT cells detected as early as 30 min and a continued increase of Atf4, with maximal expression at 6 h (Fig. 4*D*). The induction of p-eIF2α and Atf4 was eliminated with the deletion of Gcn2 in these cells. The HF treatment control showed an equivalent dependence on Gcn2 for induction of Atf4 (Fig. 4*B*). These results further support the idea that low concentrations of Gcn2iB induce the ISR *via* direct activation of Gcn2. As noted earlier for HF treatment, Gcn2iB also showed enhanced p-eIF2α even in Gcn2 KO cells, but in the Gcn2 KO cells, the induction of p-eIF2α was reduced compared to that observed in WT cells and occurred largely at the higher inhibitory Gcn2iB concentrations (Fig. 4*C*). Given these results, we chose to utilize Atf4 as the read-out for Gcn2 activity in subsequent experiments.

### RWD and HARS-related domains contribute to Gcn2 activation by HF but are dispensable for activation by Gcn2iB

**Figure 5:**
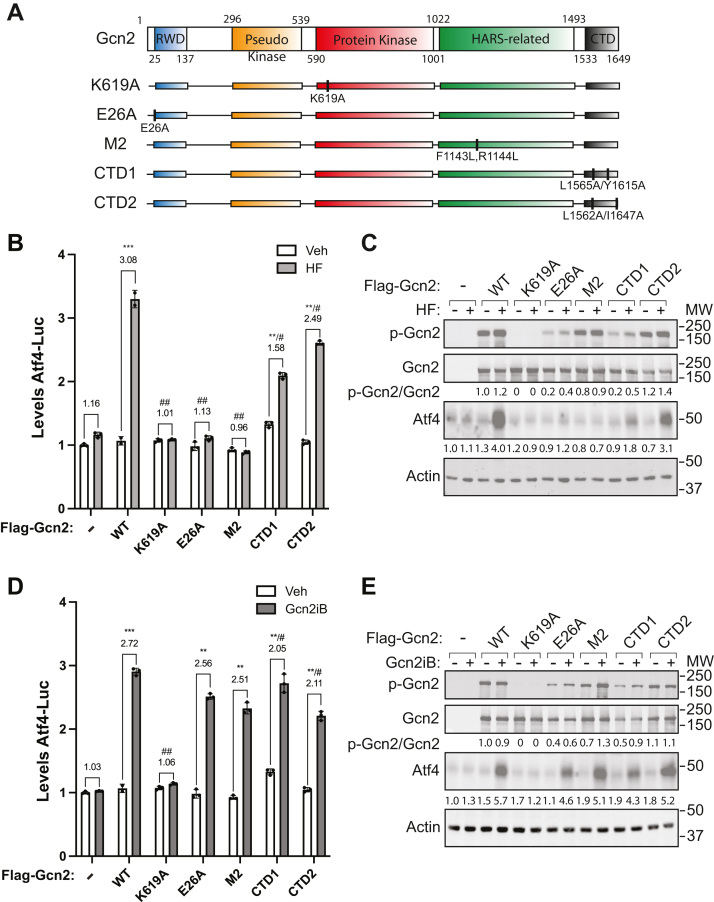
**RWD and HisRS-related domains are required for Gcn2 activation by HF but are dispensable for induction *via* Gcn2iB.***A*, The indicated residues in the regulatory domains of Gcn2 were individually substituted to address their contributions in the activation of Gcn2 and the ISR in HEK293 cells. *B*, Atf4-Luc activity was measured in HEK293 Gcn2 KO cells transiently expressing WT Gcn2 and the indicated mutant proteins. Cells were treated with 25 nM HF or vehicle for 6 h and luciferase activity was measured and represented in the bar graph normalized to empty vector–transfected Gcn2 KO cells (−) treated with vehicle. Error bars represent the SD of the mean of three biological samples, with each data point represented in the bar graph. *C*, levels of the indicated phosphorylated and total proteins were measured by immunoblot analyses in the HEK293 cells expressing WT or the Gcn2 mutants analyzed in panel *B*. *D*, Atf4-Luc activity was measured in HEK293 cells transiently expressing WT or the indicated Gcn2 mutant proteins treated with 50 nM Gcn2iB or vehicle for 6 h. Error bars represent the SD of the mean of three biological samples, with each data point represented. *E*, HEK293 cells transiently expressing WT Gcn2 or the indicated mutants from panel D were analyzed by immunoblot analyses to determine the levels of the indicated total and phosphorylated proteins. For panels *B* and *D*: the fold change upon treatment is displayed above each grouping. Welch’s *t* test was used to compare the fold change in luciferase activity upon treatment and ∗ indicates significant changes relative to the fold change of empty vector (−) transfected cells, while # indicates a significant difference relative to the fold change of WT transfected cells. # = *p* < 0.05, ∗∗ or ## = *p* < 0.01, ∗∗∗ = *p* < 0.001. HF, Halofuginone; ISR, integrated stress response.

Multiple regulatory domains are involved in the activation of the Gcn2 during stress (Fig. 5*A*). We used the Gcn2 KO cells and transient transfection of WT or mutant Flag-tagged versions of Gcn2 to address the contributions of these regulatory regions for Gcn2 activation in response to HF and Gcn2iB. The following missense mutations were introduced in *Gcn2* to specifically disrupt the function of the key domains: E26A (RWD), K619A (protein kinase), F1143L/R1144L (m2 motif in HARS), and L1565A/Y1615A and L1562A/I1647A (CTD) (Fig. 5*A*). The mutations in the encoded protein kinase and HARS-related domains are reported to block Gcn2 eIF2α kinase activity and tRNA binding, respectively (10, 42). The substitutions in the RWD and CTD regions of Gcn2 are predicted to disrupt their functions based on the reported structures of these domains (8, 43, 44). The functional consequences of these changes were determined using the Atf4-Luc translational reporter. When Gcn2 KO HEK293 cells transfected with WT or mutant Gcn2 were treated with 25 nM HF, a three-fold increase was observed in the Atf4-Luc activity in the cells expressing WT Gcn2 (Fig. 5*B*). However, there was minimal increase in the Gcn2 KO cells transfected with empty vector (−) or expressing the kinase-deficient K619A mutant, RWD E26A, or HARS M2 versions of Gcn2. The CTD1 and CTD2 mutants experienced modest induction of Atf4-Luc activity, but each was significantly less than cells expressing WT Gcn2 (Fig. 5*B*). The WT and Gcn2 mutant proteins were expressed at similar levels as judged by immunoblot (Fig. 5*C*). Consistent with these results, increased Atf4 protein levels were observed in WT cells but not in cells expressing the RWD and M2 mutant, while a moderate induction of Atf4 protein levels was observed in the CTD mutant cells treated with HF (Fig. 5*C*). These results indicate that each of the regulatory regions of Gcn2 are critical for eIF2α kinase activation in response to HF, although the CTD mutants showed some partial activities.

We next sought to evaluate the effects of these mutations in *Gcn2* on the induction of the ISR by Gcn2iB. In cells expressing WT Gcn2 treated with Gcn2iB, there was a 2.7-fold increase in Atf4-Luc expression, which was absent in the Gcn2 KO cells (Fig. 5*D*). Of importance, while cells expressing the Gcn2 kinase-deficient K619A showed no induction of Atf4-Luc activity, there was robust luciferase activity in cells with Gcn2 mutants substituted in the RWD, HARS-related, and CTD regulatory regions. Commensurate with increased *Atf4* translation, Gcn2iB increased Atf4 protein levels in cell expressing WT and the E26A, M2, CTD1, and CTD2 mutant Gcn2, but not the K619A kinase-dead mutant (Fig. 5*E*). These results indicate that while the RWD and HARS-related domains are required for Gcn2 induction by accumulating uncharged tRNA triggered by HF, these domains are dispensable for activation by Gcn2iB. Gcn2iB is likely directly engaging with the kinase domain of Gcn2 to stimulate its activity.

### Substitutions in the Gcn2 eIF2α kinase domain alter activation by HF and Gcn2iB

**Figure 6:**
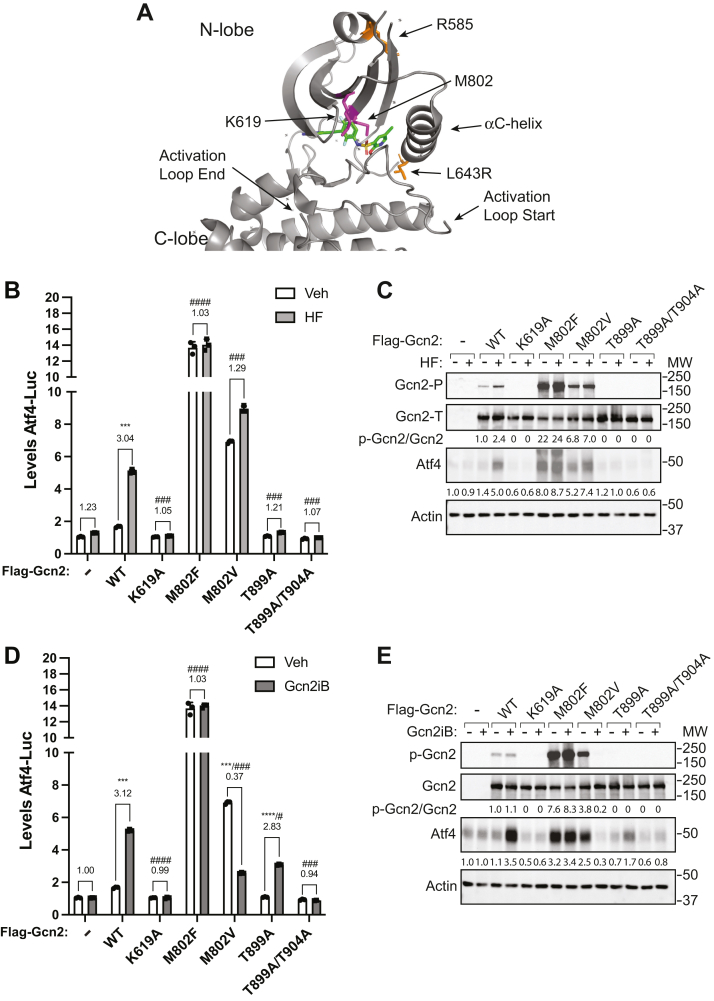
**Mutations near the ATP-binding site of the Gcn2 kinase domain alter activation by HF and Gcn2iB.***A*, structure of the GCN2 kinase domain (shown in *gray*) with bound Gcn2iB (PDB: 6N3O) (27). M802 and K619 residues are highlighted in *purple*, and R585 and L643 residues are shown in *orange*. Residues T899 and T904 are located in the activation loop, which is missing from the crystal structure due to its conformational flexibility. *B*, Atf4-Luc activity was measured in Gcn2 KO HEK293 cells transiently expressing WT or the indicated mutant Gcn2 proteins. Cells were treated with 25 nM HF or vehicle for 6 h, and the Atf4-Luc activities were determined. The Atf4-Luc levels are presented in the bar graph and are normalized to empty vector (−) vehicle-treated cells. Error bars represent the SD of the mean of three biological samples, which are each represented by data points. *C*, Gcn2 KO HEK293 cells transfected with empty vector (−) or the indicated flag-tagged Gcn2 were treated with either 25 nM HF (+) or vehicle (−) for 6 h. Levels of total and phosphorylated Gcn2 (T899), Atf4, and actin were measured by immunoblot analyses. Atf4 was normalized to empty vector–transfected vehicle-treated sample, while p-Gcn2/Gcn2 was normalized to WT-transfected cells treated with vehicle. *D*, Gcn2 KO cells transiently expressing WT or the indicated mutant Gcn2 were assayed for Atf4-Luc activity. Cells were treated with 50 nM Gcn2iB or DMSO vehicle for 6 h. The Atf4-Luc activities are presented in the bar graph normalized to empty vector–transfected Veh-treated sample. *E*, Gcn2 KO cells used in the experiment presented in panel *D* were treated with 50 nM Gcn2iB or DMSO Veh for 6 h. Levels of p-Gcn2, total Gcn2, Atf4, and actin were measured by immunoblot analyses. For *C* and *E*: The fold change upon treatment is displayed above each grouping. Welch’s *t* test was used to compare the fold change in luciferase activity upon treatment with ∗ indicating significant changes relative to empty vector (−) transfected cells and # indicating a significant difference relative to the fold change upon treatment in WT-transfected cells. # = *p* < 0.05, ∗∗∗ or ### = *p* < 0.001, ∗∗∗∗ or #### = *p* < 0.0001. HF, Halofuginone.

Based on the reported structure of the Gcn2 eIF2α kinase domain (27), we introduced residue changes in the ATP-binding pocket and the activation loop (Fig. 6*A*). The mutant versions of Gcn2 were expressed in the Atf4-Luc–expressing cells that were treated with HF or Gcn2iB to evaluate their impact on Gcn2 regulation. These Gcn2 changes included alanine substitutions to the invariant lysine residue (K619A) or both activation loop threonine residues that are subject to autophosphorylation (T899A and T899A/T904A) (45), along with phenylalanine and valine substitutions for the gatekeeper methionine residue (M802F and M802V). It was shown previously in *Saccharomyces cerevisiae* that substitution of the gatekeeper methionine residue of Gcn2 to valine leads to a moderate constitutive activation of Gcn2 (2, 46). We reasoned that since Gcn2iB binds Gcn2 in a type I ½ mode where the compound extends into a hydrophobic pocket formed by the outward rotation of the αC-helix away from the active site (27), binding of the compound may be blocked by substitution of the gatekeeper residue, that is positioned near the entry to this pocket, by a bulky residue such as phenylalanine.

In response to HF treatment, cells expressing WT Gcn2 showed a 2.7-fold increase in Atf4-Luc activity while there was no induction with the Gcn2 K619A mutant (Fig. 6*B*). Furthermore, there was no induction of Atf4-Luc activity in the HF-treated cells expressing Gcn2-containing substitutions for the autophosphorylated threonine residues, emphasizing their importance in the ordered mechanism of Gcn2 activation. However, the gatekeeper mutants M802F or M802V showed a 15-fold and 7-fold increase in Atf4-Luc expression, respectively, which occurred independent of HF treatment (Fig. 6*B*). Levels of the WT and mutant Gcn2 proteins were similar as judged by immunoblot analyses, and levels of Atf4 protein in this collection of Gcn2-expressing cells showed similar patterns of induction, with M802F and M802V cells showing pronounced increases in Atf4 protein levels even in the absence of HF treatment (Fig. 6*C*).

Cells expressing the different Gcn2 mutants were then treated with 50 nM Gcn2iB for 6 h, and those expressing WT Gcn2 had a 3-fold increase in Atf4-Luc activity, whereas cells transfected with empty vector (−) or expressing kinase-deficient Gcn2 (K619A) had no increase in Atf4-Luc (Fig. 6*D*). Cells containing Gcn2 M802F showed a large induction of Atf4-Luc activity in the presence or absence of Gcn2iB. Of interest, Gcn2 M802V also showed enhanced Atf4-Luc activity, but there was a sharp reduction with Gcn2iB treatment, indicative of inhibition rather than stimulation of Gcn2 activity (Fig. 6*D*). Furthermore, cells expressing Gcn2 T899A showed a 2.8-fold increase in luciferase activity upon Gcn2iB treatment, although it should be emphasized that the basal levels of Atf4-Luc expression were lower than WT and comparable to vector. The Gcn2 autophosphorylation mutant T899A/T904A did not display any increase in Atf4-Luc activity (Fig. 6*D*). Levels of Gcn2 were similar between WT, and these kinase-domain mutants of Gcn2 and the Atf4 protein levels showed a similar pattern of induction as described for the Atf4-Luc reporter (Fig. 6*E*). These results indicate that M802F and M802V substitutions cause an induction of Gcn2 kinase activity in the absence of stress. Although Gcn2iB has no effect on Gcn2 M802F, 50 nM Gcn2iB led to inhibition rather than activation of Gcn2 M802V. Furthermore, autophosphorylation of T899 in the activation loop of Gcn2 is required for induction of the ISR by HF. By contrast, phosphorylation of T899 is at least partially dispensable for activation by Gcn2iB, although Gcn2iB no longer activated Gcn2 when phosphorylation of both T899 and T904 were blocked. Together, these results are consistent with a model where binding of Gcn2iB contributes to a catalytically active conformation, bypassing some steps in the ordered mechanism of Gcn2 activation.

### Activation of Gcn2 by gatekeeper substitution and kinase inhibitor binding

**Figure 7:**
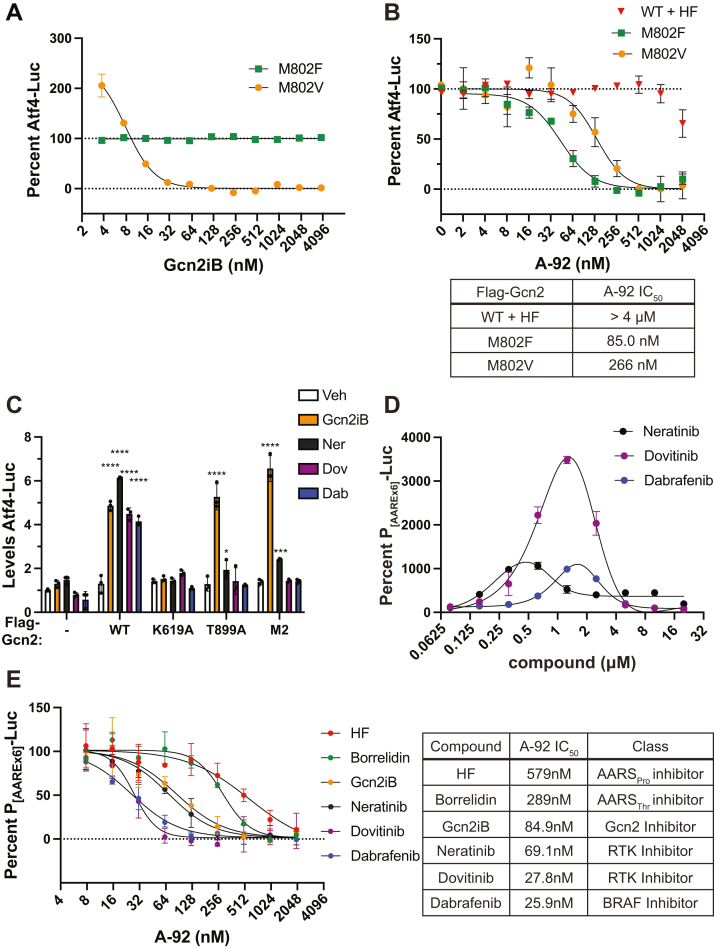
**Activation of Gcn2 by gatekeeper substitution and kinase inhibitor binding.***A*, Atf4-Luc activity was measured in Gcn2 KO HEK293 cells expressing M802V or M802F mutant versions of Gcn2. Cells were treated with the indicated concentration of Gcn2iB or vehicle for 6 h. Atf4-Luc is expressed as a percentage relative to untreated cells. Error bars represent the SD of three biological replicates. *B*, Levels of Atf4-Luc were measured in cells expressing WT, M802V, or M802F versions of Gcn2. WT Gcn2 expressing cells were pretreated with the indicated concentration of A-92 for 30 min and then 25 nM HF was added to the medium for an additional 6 h. In parallel, cells expressing Gcn2 M802F or M802V were treated with only A-92 for 6 h. Levels of Atf4-Luc are presented as a percentage relative to untreated cells (for M802F and M802V) or cells treated with HF only (for WT Gcn2). Error bars represent the SD of three biological replicates. The IC_50_ values for A-92 were calculated using variable slope (four parameter) nonlinear regression. Values for IC_50_ are displayed in the table below the graph. *C*, Gcn2 KO HEK293 cells were transfected with empty vector (−), WT, K619A, T899A, or M2 Gcn2. Cells were treated with vehicle, 50 nM Gcn2iB, 1 μM neratinib, 2 μM dovitinib, or 2 μM dabrafenib for 6 h and then Atf4-Luc activity was measured and presented normalized to vehicle-treated cells with empty vector (−). Error bars represent the SD of three biological replicates, which are each represented by points. Significant changes in Atf4-Luc activity were measured by two-way ANOVA comparing each treatment to the vehicle for that genotype using Dunnett correction for multiple hypothesis testing: ∗ = Adj. *p* < 0.05, ∗∗∗ = *p* < 0.001, ∗∗∗∗ = *p* < 0.0001. *D*, HEK293 cells containing the integrated P_(AAREx6)_-Luc reporter were treated with the indicated concentrations of Dovitinib or Neratinib for 6 h, and luciferase activity was measured and is presented as a percentage of Veh-treated luciferase activity. Error bars represent the SD of three biological samples. The overlaid curves were generated using Bell-shaped nonlinear regression in GraphPad Prism, where x is concentration and y is luciferase activity. *E*, cells expressing endogenous Gcn2 and P_(AAREx6)_-Luc were pretreated with the indicated concentrations of A-92 for 30 min and then treated with 12.5 nM halofuginone, 1 μM borrelidin, 31.25 nM Gcn2iB, 1 μM neratinib, 2 μM dovitinib, or 2 μM dabrafenib for 6 h. Luciferase activity was measured and is expressed as a percentage relative to cells treated with agonist only. Error bars represent the SD of three biological replicates. IC_50_ values for inhibition by A-92 were calculated using variable slope (four parameter) nonlinear regression and are displayed in the table to the right. HF, Halofuginone.

Given that the gatekeeper M802F and M802V substitutions constitutively enhance Gcn2 activity and altered induction in response to 50 nM Gcn2iB treatment, we carried out a dose-response curve for Gcn2iB up to an inhibitory concentration of 4 μM (Fig. 7*A*). Here, Atf4-Luc activity is expressed as a percentage of activity of untreated cells, and as noted previously, cells expressing M802F or M802V each display much greater Atf4-Luc activity than untreated cells transfected with WT Gcn2. Cells expressing Gcn2 M802F maintained the same level of Atf4-Luc activity even with Gcn2iB concentrations up to 4 μM (Fig. 7*A*). By comparison, cells with Gcn2 M802V displayed a 2-fold increase in Atf4-Luc activity at 4 nM Gcn2iB treatment relative to untreated M802V cells. Increasing concentrations of Gcn2iB led to a rapid reduction in Atf4-Luc signal until a minimum was reached between 50 and 100 nM Gcn2iB that was sustained until 4 μM (Fig. 7*A*). These results are in keeping with our observation that 50 nM Gcn2iB had an inhibitory effect on ISR activation in cells transfected with M802V Gcn2 plasmid. Rather than acting solely as an inhibitor of Gcn2 M802V, the biphasic response observed upon increasing Gcn2iB amounts has been shifted to the left, toward lower concentrations, suggesting tighter Gcn2 binding with Gcn2iB.

We next probed the ATP-binding properties of Gcn2 M802F and M802V by substituting the type I ATP-competitive Gcn2 inhibitor A-92 for Gcn2iB in a similar dose-response experiment. While M802F and M802V displayed high Atf4-Luc activity in the absence of stress, Gcn2 WT cells were activated with 25 nM HF to allow for measurable inhibition by A-92. Gcn2 WT, M802F, or M802V cells were each treated with increasing A-92 concentrations, and Atf4-Luc was measured to assess Gcn2 activity (Fig. 7*B*). The IC_50_ values calculated for inhibition of Gcn2 by A-92 show a dramatic reduction in Gcn2 M802F and M802V activities when compared to Gcn2 WT, suggesting an increased binding affinity for A-92 with each of the Gcn2 gatekeeper mutants (Fig. 7*B*).

It was previously reported that the receptor tyrosine kinase inhibitors neratinib and dovitinib can activate Gcn2 (29). Dabrafenib is a BRAF inhibitor that was previously reported to paradoxically activate RAF–MEK–ERK signaling axis (30) and is also suggested to activate Gcn2 (47). To compare the activation of Gcn2 by these different kinase inhibitors, we measured Gcn2 activation upon treatment with each using the Atf4-Luc reporter. Gcn2 KO HEK293 cells were transfected with empty vector (−), WT, K619A, T899A, and M2 Gcn2 plasmids and then treated with either 50 nM Gcn2iB, 1 μM neratinib, 2 μM dovitinib, or 2 μM dabrafenib, and Atf4-Luc activity was measured after 6 h (Fig. 7*C*). Atf4-Luc activity increased similarly upon treatment with each compound, between 3- to 5-fold, in cells expressing WT Gcn2, while no increase in Atf4-Luc activity was observed in cells expressing the catalytically inactive K619A mutant of Gcn2. In cells expressing Gcn2 T899A or M2, only Gcn2iB and neratinib were able to increase Atf4-Luc activity, though the extent of the increase by Gcn2iB was much greater than that of neratinib (Fig. 7*C*). Therefore, while each compound induced similar Atf4-Luc activity in WT Gcn2 cells, only Gcn2iB and to a lesser extent neratinib showed significant enhanced Atf4 translational expression in cells expressing Gcn2 T899A and M2 mutants. These results suggest that there are important differences in the mechanisms by which these compounds can activate Gcn2. These differences include the ability of Gcn2iB to activate Gcn2 *via* processes that do not require autophosphorylation at T899.

As we previously observed that Gcn2iB produced a biphasic induction of Atf4 transcriptional activity (Fig. 3*B*), we sought to measure the effect of compound dose of neratinib, dovitinib, and dabrafenib for induction of the ISR. HEK293 cells stably expressing P_(AAREx6)_-Luc reporter were treated with a 2× serial dilution from 78 nM to 20 μM for each compound (Fig. 7*D*). As described for Gcn2iB (Fig. 2*B*), each of these compounds elicited a biphasic induction of Atf4 activity as measured by the P_(AAREx6)_-Luc reporter (Fig. 7*D*). Therefore, Gcn2iB, neratinib, dovitinib, and dabrafenib each exhibit a similar induction of the ISR, although the peak concentration and magnitude varied, likely reflecting differences in Gcn2-binding affinity between the compounds.

Given that A-92 produced different IC_50_ values with Gcn2 activated by HF *versus* Gcn2iB (Fig. 3*A*), we determined whether this difference also held true for the other compounds. We measured the effects of A-92 following activation of Gcn2 by borrelidin, a potent inhibitor of aminoacylation of tRNA^Thr^ (48, 49), HF, Gcn2iB, neratinib, dovitinib, or dabrafenib. Gcn2 WT cells expressing the integrated P_(AAREx6)_-Luc reporter were treated individually with these compounds in combination with increasing doses A-92 for 6 h. When Gcn2 was induced by Gcn2iB, neratinib, dovitinib, or dabrafenib, IC_50_ values for A-92 were less than 100 nM, whereas with borrelidin or HF, IC_50_ values were determined to be 289 nM and 579 nM, respectively (Fig. 7*E*). Thus, Gcn2 inhibition by A-92 is more potent when a kinase inhibitor is used as a Gcn2 agonist compared to when this eIF2α kinase is activated by agents that induce uncharged tRNAs. These results suggest that there are distinct mechanisms by which ATP-competitive kinase inhibitors can induce Gcn2 activity: one mechanism that requires Gcn2 phosphorylation of the activation loop T899, as with neratinib and dovitinib, and another mechanism, exemplified by Gcn2iB, that does not depend on T899 phosphorylation.

### Gcn2iB activation of PVOD causing Gcn2 mutants

**Figure 8:**
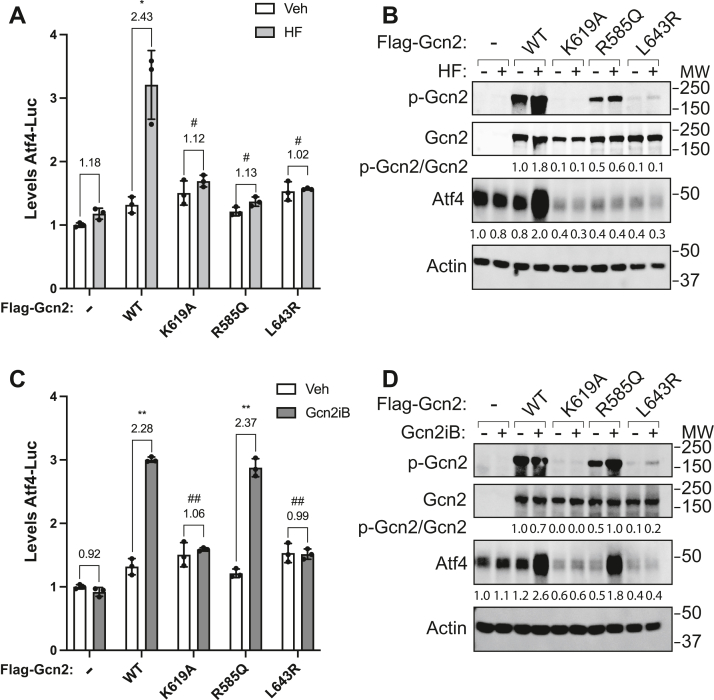
**Gcn2 mutations reported in PVOD patients are activated by Gcn2iB.***A*, HEK293 Gcn2 KO cells transiently expressing the indicated WT or mutant versions of Gcn2, or empty vector (−), were treated with 25 nM HF or vehicle for 6 h. Levels of Atf4-Luc were measured and are presented normalized to empty vector (−) Veh-treated sample. Error bars represent the SD of the mean of three biological samples, which are each represented as data points on the bar graph. *B*, HEK293 Gcn2 KO from panel *A* were treated with 25 nM HF for 6 h, and the levels of the indicated total and phosphorylated proteins were measured by immunoblot. Levels of p-Gcn2 and Atf4 are presented relative to vehicle-treated cells transfected only with vector. *C*, Gcn2 KO HEK cells expressing WT or the indicated mutant versions of Gcn2, or empty vector, were treated with 50 nM Gcn2iB or vehicle (Veh) for 6 h. Atf4-Luc expression was measured and presented in the bar graph. Error bars represent the SD of the mean of three biological samples, which are each represented by datapoints. The fold change upon treatment is displayed above each experimental group. *D*, HEK293 Gcn2 KO cells were transfected with flag-tagged WT or mutant version of Gcn2, or vector alone (−), as indicated. Cells were treated with either 50 nM Gcn2iB (+) or vehicle (−) for 6 h. The indicated total and phosphorylated proteins were measured by immunoblot analyses. Quantitation for p-Gcn2 and Atf4 are shown below each of the respective blots. Phosphorylated Gcn2 was normalized to WT vehicle, and Atf4 was normalized to empty vector (−) Veh-treated cells. For *A* and *C*: Welch’s *t* test was used to compare the fold change in luciferase activity upon treatment with ∗ indicating significant changes relative to the fold change of empty vector (−) transfected cells and # indicating a significant difference relative to the fold change upon treatment in WT-transfected cells. ∗ or # = *p* < 0.05, ∗∗ or ## = *p* < 0.01. HF, Halofuginone; PVOD, pulmonary veno-occlusive disease.

It was previously reported that missense mutations R585Q and L643R within the kinase domain of Gcn2 contribute to PVOD (21). Residue R585 is located in the αA-helix, and L643R is located in the loop between the αC-helix and the β4-strand (Fig. 4*A*). We expressed Gcn2 WT or these variants in Gcn2 KO cells and determined that the Gcn2 R585Q and L643R substitutions blocked both induction of Atf4-Luc activity and increased Atf4 protein levels in response to HF treatment (Fig. 8, *A* and *B*). There were similar levels of Gcn2 WT, R585Q, and L643R proteins as measured by immunoblot (Fig. 8*B*). However, it is noted that there was some autophosphorylation of Gcn2 R585Q, albeit at reduced levels as compared to Gcn2 WT. These results indicate that the Gcn2 substitutions derived from PVOD patients prevent Gcn2 activation by accumulating uncharged tRNA.

We next determined whether the Gcn2 R585Q and L643R substitutions were activated by Gcn2iB treatment. Interestingly, Gcn2 WT and R585Q cells showed similar induction of Atf4-Luc activity upon Gcn2iB treatment, whereas cells expressing Gcn2 L643R were devoid of activity (Fig. 8*C*). The Gcn2 mutant proteins were expressed at similar levels as WT. Gcn2iB induced Atf4 protein levels in Gcn2 WT and R585Q cells as judged by immunoblot to a similar degree, while no increase was observed in vector control, K619A, or L643R mutant-expressing cells (Fig. 8*D*). These results indicate that Gcn2iB is able to rescue the activity of certain mutant Gcn2 proteins expressed in PVOD patients.

## Discussion

Recent studies have described pharmacological strategies for inhibiting Gcn2 to treat a range of diseases, including cancers and neurological disorders (22, 23, 24, 26). Here, we report that low concentrations of the Gcn2 inhibitor Gcn2iB can activate the ISR in the absence of environmental stress by a mechanism that depends on induced Gcn2 phosphorylation of eIF2α (Figs. 3, *A*–*C*, 4, *C* and *D*, and S1). Gcn2iB activation of Gcn2 can bypass key regulatory domains required for the activation of this eIF2α kinase during stress. For example, Gcn2iB enhanced the ISR as exemplified by *Atf4* translational expression in cells expressing the Gcn2 M2 mutant, which is reported to block binding with uncharged tRNAs, or with key substitutions in the RWD, suggested to be critical for Gcn2 association with Gcn1, but not Gcn2 K619A that ablates eIF2α kinase activity (Fig. 5, *B*–*E*). Furthermore, autophosphorylation of T899 in the activation loop of the kinase domain of Gcn2 is dispensable for activation by Gcn2iB (Fig. 6, *B*–*E*). We also provide evidence that PVOD disease–causing substitutions L643R and R585Q in the Gcn2 kinase domain impair Gcn2 activation by accumulation of uncharged tRNAs invoked by HF treatment, but that R585Q Gcn2 can be activated by Gcn2iB (Fig. 8, *A*–*D*). These results show a potential therapeutic utility of pharmacologically activating Gcn2 with small molecules when the kinase domain of Gcn2 retains the capacity for substrate phosphorylation but where its regulation is disrupted. Furthermore, our study highlights the importance of the appropriate dose of eIF2α kinase inhibitors in therapeutic applications. At high concentrations, Gcn2iB is an effective inhibitor of Gcn2 kinase activity, whereas at lower concentrations, Gcn2iB is an activator of the eIF2α kinase.

### Gcn2 activation by compounds designed to be protein kinase inhibitors

Previous studies have suggested an ordered mechanism of Gcn2 activation involving multiple regulatory domains that monitor for nutrient limitations and other environmental stresses. The HARS-related domain binding to uncharged tRNAs that accumulate during amino acid depletion (10, 11, 42), the RWD association with Gcn1 that has been proposed to facilitate Gcn2 access to uncharged tRNAs (50) and Gcn2 engagement with colliding ribosomes (6), and the CTD that is reported to contribute to dimerization and Gcn2 binding to ribosomes that is critical for Gcn2-sensing environmental stresses (7, 8, 9) are all suggested to trigger conformational changes in Gcn2 that involve back-to-back dimerization of the protein kinase domain, transphosphorylation of activation loop residues, and substrate binding. The process by which Gcn2iB activates Gcn2 appears to bypass key events involving the Gcn2 regulatory domains. These results are consistent with a report by Kato *et al* (51) that suggested that a truncated recombinant Gcn2 protein retaining the protein kinase but devoid of some regulatory domains can be activated for eIF2α phosphorylation by Gcn2iB. Furthermore, additional type I ½ compounds designed to inhibit Gcn2 have been suggested to activate this eIF2α kinase at low concentrations, further supporting the underlying concepts described in this study (52).

The data presented here support a previously proposed model, wherein binding of an ATP-competitive inhibitor to one protomer of the kinase domain dimer, such as Gcn2, can lead to activation of the second protomer (29). In this model, Gcn2iB engages with the ATP-binding pocket of the Gcn2 protein kinase domain. Phenylalanine substitution of the gatekeeper M802, which is situated at the back of the ATP-binding pocket, is suggested to block Gcn2iB binding and thwarts regulation by this compound (Fig. 7*A*). Furthermore, A-92 effectively competes with Gcn2iB, thereby negating Gcn2iB activation of Gcn2 (Figs. 3, *A* and *B* and 7, *B* and *E*). Gcn2iB activation can occur even in the absence of phosphorylation of the activation loop site T899, which suggests that mere binding of Gcn2iB can induce certain conformations that are analogous to those triggered by Gcn2 autophosphorylation. It was previously reported that substitution of the yeast Gcn2 L856 residue (human Gcn2 L869), and the resulting disruption of hydrophobic interactions within the back pocket of the ATP-binding cleft, led to constitutive activation of Gcn2 that similarly bypassed the requirement for activation loop phosphorylation by a mechanism suggested to facilitate the inward rotation of the αC-helix (53). In the same study, it was also noted that substitutions along the kinase dimer interface, shown previously to enhance dimerization and activity of the ISR protein kinase Pkr (54), also constitutively activated Gcn2 independent of activation loop phosphorylation. It is also noteworthy that a type I ½ inhibitor of PERK has been shown to induce PERK kinase domain dimerization (55). Thus, Gcn2iB binding to the ATP pocket of one protomer potentially leads to an active conformation of the adjacent Gcn2 protein kinase domain promoter, possibly through enhanced Gcn2 kinase domain dimerization, or through disruption of inhibitory interactions within the hydrophobic back pocket of the ATP-binding site, or a combination of both.

Several other type I ½ ATP-competitive inhibitors, including neratinib, dovitinib, and dabrafenib, also activated Gcn2 and the ISR (Fig. 7, *C* and *D*). Activation of Gcn2 by these compounds is likely to occur by binding to the ATP pocket of Gcn2 by processes similar to that for Gcn2iB. Analogous to Gcn2iB, activation of Gcn2 by neratinib, dovitinib, or dabrafenib was blocked by A-92, suggesting that the binding of these compounds to Gcn2 triggers conformations conducive to activation (Fig. 7*E*). However, there appears to be important differences in the activation mechanisms between Gcn2iB and these other type I ½ inhibitors. Only Gcn2iB was observed to activate Gcn2 M2, and neratinib, dovitinib, and dabrafenib also required T899 and the attendant autophosphorylation (Fig. 7*C*). These results suggest multiple related mechanisms by which type I ½-half inhibitors can activate Gcn2, and these mechanistic differences can dictate which steps in the ordered mechanism of Gcn2 activation are effectively bypassed.

### Utility of Gcn2-activating compounds

Gcn2 has a primary adaptive function to nutrient stress, and loss of Gcn2 enhances morbidity in mice-fed amino acid–deficient diets (1). In certain cancer models, Gcn2 is critical for uptake and synthesis of amino acids, and genetic or pharmacological depletion of the eIF2α kinase blocks progression in cultured cell and xenograft models (24, 26). The adaptive function of Gcn2 in cancers was a central reason for the discovery and development of Gcn2iB as an inhibitor of Gcn2 in single or combination anticancer therapies. While Gcn2 and the ISR can serve critical adaptive functions in response to acute stresses, during chronic stresses, the ISR can be maladaptive. The maladaptive Gcn2 function is illustrated in CMT, where underlying mutations in aminoacyl tRNA synthetase genes result in elevated levels of uncharged tRNAs and ribosome stalling that are suggested to chronically activate Gcn2 leading to injury to peripheral nerve tissue. Inhibition of Gcn2 or the ISR was reported to alleviate the peripheral neuropathy of CMT (22, 23).

While inhibition of Gcn2 can provide therapeutic benefit, there are other medical conditions where activation of this eIF2α kinase and the ISR have utility. In the context of glioblastoma, activation of Gcn2 by EGFR inhibitors was suggested to have a synergistic effect in reducing cancer cell survival and proliferation (29). Loss of Gcn2 function leads to pulmonary disorders (18, 19, 20, 21), and we showed that low concentrations of Gcn2iB can activate Gcn2 R585Q mutant derived from a PVOD patient (Fig. 8, *C* and *D*), which offers new therapeutic strategies for treatment of those expressing missense or truncated Gcn2 mutant proteins that retain an intact protein kinase domain. Another avenue for pharmacological activation of Gcn2 involves induction of the ISR prior to a major stress, such as surgery. It was reported that dietary amino acid restriction or treatment with HF conferred resistance in models of surgical ischemia injury (56). The rationale for this protection is that invoking the protective gene expression of the ISR would better prepare tissues against subsequent stress injury. In this regard, compounds that activate Gcn2 could be applied for the preconditioning benefit to alleviate tissue injury.

## Experimental procedures

### Cell culture and generation of cell lines

HEK-derived cells were used in this study. HEK293T cells were obtained from American Type Culture Collection. HEK293A cells were purchased from Invitrogen. Cells were cultured in Dulbecco's modified Eagle's medium (DMEM) (Corning 10-013-CV) supplemented with 10% (vol/vol) fetal bovine serum (Corning 35-010-CV), 100 U/ml penicillin, and 0.1 mg/ml streptomycin (Hyclone SV30010), hereafter referred to as DMEM complete, at 37 °C in 5% CO_2_ humidified incubator. Treatment doses and times for HF (Cayman Chemical Co. #13370), Gcn2iB (Med. Chem. Express #HY-112654), A-92 (Med. Chem. Express #HY-100877), 2B-Act (Med. Chem. Express #HY-125021), dabrafenib (SelleckChem #52807), borrelidin (Tocris #4706), neratinib (Med. Chem. Express #HY-32721), and dovitinib (Med. Chem. Express #HY-50905) are indicated in the descriptions of experiments. To create cells stably expressing a luciferase reporter for Atf4 transcription activity, HEK293A cells were transduced with a lentivirus encoding an Atf4 transcription reporter (P_(AAREx6)_-Luc), which consists of six copies of C/ebp-Atf4 consensus binding sequence (5′-AACATTGCATCATCCCCGC-3′) upstream of a minimal promoter followed by the firefly luciferase coding sequence. Selection of the stable expression of the luciferase reporter was selected in DMEM complete supplemented with 1 μg/ml puromycin.

Deletion of Gcn2 in HEK-derived cells was achieved through use of a CRISPR/Cas9 Human Gene Knockout Kit (Origene Cat# KN412459). Briefly, 0.2 × 10^6^ cells were seeded into 6-well TC plates, and 1 μg of a plasmid pCas-Guide-Gcn2 plasmid encoding Cas9 and a sgRNA (5′- GGACTTCCAAGACCTGCGGC-3′) were transfected using FuGENE 6 reagent into the HEK-derived cells. Transfections were carried out for 24 h with 3:1 ratio of FuGENE to plasmid DNA. The transfected cells were passaged five times over the course of 10 days, and cells were then seeded into 10 cm plates at a density of 200 cells/plate to allow for colony formation. Cells were maintained DMEM complete for 7 to 10 days with media change every other day until colonies of cells could be easily identified. The colonies were then expanded and screened for Gcn2 expression by immunoblot analysis. The Gcn2 KO cells were confirmed by sequencing of the genomic DNA segment of the Gcn2 gene and by the loss of expressed Gcn2 protein.

### Immunoblot analyses

HEK-derived cells were cultured in DMEM as described in the Cell culture methods. Lysates were prepared from the HEK-derived cells using a solution containing 1% SDS, 20 mM Tris (pH 7.5), and 150 mM NaCl supplemented with 1× Halt protease and phosphatase inhibitor cocktail (Thermo Fisher Scientific 1861281). Cell lysates were boiled for 5 min, then sonicated using a Branson sonifier, and clarified by centrifugation at 12,000*g*. Protein concentrations were measured using a DC protein assay kit (Bio-Rad #5000112) with bovine serum albumin as a standard. Equal amounts of protein lysates were separated by electrophoresis in SDS gels and transferred onto 0.2 μm nitrocellulose membranes using a Trans-Blot Turbo RTA nitrocellulose transfer kit (Bio-Rad #1704270). The protein-bound membranes were incubated with primary antibodies in TBS-T solution (50 mM Tris pH 7.5, 150 mM NaCl, 0.2% Tween-20) at room temperature with gentle agitation. The following primary antibodies were used in the immunoblot analyses: p-Gcn2-T899 1:500 (Abcam #75836), total Gcn2 1:1000 (Cell Signaling #3302), p-eIF2α-S51 1:1000 (Abcam #32157), total eIF2α 1:2000 (Cell Signaling #5342), Atf4 1:1000 (Cell Signaling #11815), actin 1:3000 (Sigma #A5441). The membranes were washed three times for 10 minutes with TBS-T solution and then incubated with 1:3000 GAR-HRP (Bio-Rad #170-6515) or GAM-HRP (Bio-Rad #170-6516) secondary antibodies for 45 min. The membranes were washed for three times with TBS-T solution, each for 10 minutes. Immunoblot signals were visualized with ECL solution (1:1 mixture of 100 mM Tris pH 8.5, 2.5 mM luminol, 0.4 mM p-coumaric acid: 100 mM Tris pH 8.5, and 0.06% H_2_O_2_ vol/vol) or SuperSignal West Femto Maximum Sensitivity Substrate (Thermo Fisher Scientific #34094), and images were visualized and saved using a Chemidoc MP imaging system (Bio-Rad).

### Plasmids

Plasmid pCDNA5 was purchased from Invitrogen (Cat #V601020). Plasmid pGL3-basic was obtained from Promega (Cat #E1751). The 5′-leader of the encoded *Mus musculus Atf4* mRNA was inserted into the pCDNA5 vector between the CMV promoter and the firefly luciferase coding sequence from pGL3-basic using an In-Fusion HD cloning kit. The resulting translation reporter plasmid is referred to in this text as Atf4-Luc.

The human Gcn2 coding sequence from pENTR223-1 Gcn2 (NM_001013703; Transomic Technologies) was inserted into pCDNA5 to create an amino-terminal 3× flag tag fusion with Gcn2 by using an In-Fusion HD cloning kit and a three-way cloning reaction method. The resulting plasmid pCDNA5-Gcn2, referred to in the text as Flag-Gcn2, was used as a template to construct the mutant versions detailed in Table 1 by site-directed mutagenesis using an In-Fusion HD cloning kit (Takara Bio). The Gcn2 expressing plasmid was introduced transiently into the HEK293-Gcn2 KO cells as described in the following complementation section. Plasmid pMSCV-Gadd34-puro contains the human Gadd34 (PPP1R15A, NM_014330.5) gene subcloned into the NcoI/EcoRI sites of the retroviral vector pMSCV-puro (24).

**Table 1:** Flag-Gcn2 expression plasmids and their codon substitutions

Plasmid name	Codon substitution
pCDNA5-FRT-Gcn2-E26A	E26A (GAG -> GCC)
pCDNA5-FRT-Gcn2-M2	F1143L/R1144L (TCC/AGG -> CTG/CTG)
pCDNA5-FRT-Gcn2-CTD1	L1565A/Y1615A (TTA/TAC -> GCC/GCC)
pCDNA5-FRT-Gcn2-CTD2	L1562A/I1647A (CTT/ATC -> GCC/GCC)
pCDNA5-FRT-Gcn2-K619A	K619A (AAG -> GCC)
pCDNA5-FRT-Gcn2-M802F	M802F (ATG -> TTC)
pCDNA5-FRT-Gcn2-M802V	M802V (ATG -> GTG)
pCDNA5-FRT-Gcn2-T899A	T899A (ACT -> GCC)
pCDNA5-FRT-Gcn2-T899A/T904A	T899A/T904A (ACT/ACT -> GCC/GCC)
pCDNA5-FRT-Gcn2-R585Q	R585Q (CGA -> CAG)
pCDNA5-FRT-Gcn2-L643R	L643R (CTG -> CGT)

### Gcn2 complementation assays

To rescue Gcn2 function in the HEK293-Gcn2 KO cells, the KO cells were first seeded into 6 cm plates at 0.75 × 10^6^ cells/well and cultured in DMEM complete at 37 °C. The following day, cells were transfected with 750 ng of the indicated Flag-Gcn2 plasmid or pCDNA5 vector using lipofectamine 3000 transfection reagent (Invitrogen Cat # L3000008) using a 3:1 ratio of lipofectamine 3000 to plasmid DNA and a 2:1 ratio of P3000 reagent to plasmid DNA following the manufacturer's protocol. Cells were cultured for an additional 24 h in DMEM at 37 °C and then passaged into 6-well plates at a density of 0.35 × 10^6^ cells/well and cultured for 24 h. Cells were then treated with the indicated dose of compounds in DMEM for 6 h, and protein lysates were collected for immunoblot measurements of Gcn2 and other ISR biomarkers by immunoblot analyses, as described above. Alternatively, the transfected cells were assayed for luciferase assay as described in the Luciferase assay section below. A control experiment involving a mock transfection of Gcn2 WT and KO cells was carried out to measure p-eIF2α, p-Gcn2, and Atf4 levels. This experiment established that there was appreciable p-eIF2α in the KO during a time course of HF treatment.

### Luciferase reporter assays

For transcriptional reporter assays, HEK293-P_(AAREx6)_-Luc cells were seeded into 96-well plate at 10,000 cells/well and cultured in DMEM complete at 37 °C with 5% CO2 overnight. After 24 h, cells were treated with HF or Gcn2iB, as indicated. After 6 h of culture, the media was replaced with 1:1 dilution of DMEM complete to Bio-Glo luciferase assay reagent (Promega Cat # G7940). The assay plate was incubated at room temperature for 15 min at 200 rpm on rotary plate shaker and then luminescence was read on BioTek Synergy H1 microplate reader using 1 s integration time and gain setting between 135 and 200. Luciferase activity was measured as luminescence for each of three experiments per treatment.

For translational reporter assays, HEK293-Gcn2 KO cells were cotransfected with WT or mutant Gcn2 and the reporter Atf4-Luc and nano-luciferase plasmids and then treated with HF or Gcn2iB. Gcn2 activity was measured in a dual luciferase assay. To perform this experiment, HEK293-Gcn2 KO cells were seeded into 12-well plates at 50,000 cells/well. After 24 h, each well was cotransfected with either 100 ng Flag-Gcn2 or pCDNA5 empty vector, along with 100 ng Atf4-Luc and 1 ng nano-luciferase plasmids using a 3:1 ratio of FuGENE 6 transfection reagent (Promega Cat #E2692) to plasmid DNA. For experiments with Gadd34 expression, 100 ng of vector control or Gadd34 expression plasmid was included in the transfection. Cells were cultured at 37 °C in DMEM complete media for 24 h following transfection and then treated with DMSO Vehicle, 25 nM HF, or 50 nM Gcn2iB, as indicated, for 3 or 6 h. Cells were washed with PBS solution and lysed by addition of 200 μl 1× Passive Lysis Buffer (Promega Cat #E1941) followed by incubation on plate shaker at 200 rpm for 15 min at room temperature. The Atf4-Luc and nano-luciferase signals were measured with Nano-Glo Dual-Luciferase Reporter Assay kit (Promega Cat #N1620) using a Turner Biosystems 20/20 luminometer. Firefly luciferase activity was normalized to nano-luciferase control for each of three biological replicates.

## Data availability

Data is presented within the manuscript and plasmids and other reagents are available for academic purposes upon request.

## Supporting information

This article contains supporting information.

## Conflict of interest

R. C. W. is a member of the advisory board in HiberCell, Inc; K. A. S. consults for and receives research support from HiberCell, Inc. F. T. is an employee of HiberCell, Inc and is an author of patent WIPO Patent Application WO/2021/222147 that relates to Gcn2 modulation in the treatment of cancers and other diseases and disorders associated with Gcn2 activation. All other authors declare that they have no conflicts of interest with the contents of this article.

## References

[1] Wek R.C. (2018). Role of eIF2α kinases in translational control and adaptation to cellular stress. Cold Spring Harb. Perspect. Biol..

[2] Deng J., Harding H.P., Raught B., Gingras A.C., Berlanga J.J., Scheuner D. (2002). Activation of GCN2 in UV-irradiated cells inhibits translation. Curr. Biol..

[3] Jiang H.Y., Wek R.C. (2005). GCN2 phosphorylation of eIF2alpha activates NF-kappaB in response to UV irradiation. Biochem. J..

[4] Yan L.L., Zaher H.S. (2021). Ribosome quality control antagonizes the activation of the integrated stress response on colliding ribosomes. Mol. Cell.

[5] Ishimura R., Nagy G., Dotu I., Chuang J.H., Ackerman S.L. (2016). Activation of GCN2 kinase by ribosome stalling links translation elongation with translation initiation. Elife.

[6] Wu C.C., Peterson A., Zinshteyn B., Regot S., Green R. (2020). Ribosome collisions trigger general stress responses to regulate cell fate. Cell.

[7] Zhu S., Wek R.C. (1998). Ribosome-binding domain of eukaryotic initiation factor-2 kinase GCN2 facilitates translation control. J. Biol. Chem..

[8] He H., Singh I., Wek S.A., Dey S., Baird T.D., Wek R.C. (2014). Crystal structures of GCN2 protein kinase C-terminal domains suggest regulatory differences in yeast and mammals. J. Biol. Chem..

[9] Ramirez M., Wek R.C., Hinnebusch A.G. (1991). Ribosome association of GCN2 protein kinase, a translational activator of the GCN4 gene of *Saccharomyces cerevisiae*. Mol. Cell Biol..

[10] Wek S.A., Zhu S., Wek R.C. (1995). The histidyl-tRNA synthetase-related sequence in the eIF-2 alpha protein kinase GCN2 interacts with tRNA and is required for activation in response to starvation for different amino acids. Mol. Cell Biol..

[11] Dong J., Qiu H., Garcia-Barrio M., Anderson J., Hinnebusch A.G. (2000). Uncharged tRNA activates GCN2 by displacing the protein kinase moiety from a bipartite tRNA-binding domain. Mol. Cell.

[12] Garcia-Barrio M., Dong J., Ufano S., Hinnebusch A.G. (2000). Association of GCN1-GCN20 regulatory complex with the N-terminus of eIF2alpha kinase GCN2 is required for GCN2 activation. EMBO J..

[13] Marton M.J., Crouch D., Hinnebusch A.G. (1993). GCN1, a translational activator of GCN4 in Saccharomyces cerevisiae, is required for phosphorylation of eukaryotic translation initiation factor 2 by protein kinase GCN2. Mol. Cell Biol..

[14] Kubota H., Sakaki Y., Ito T. (2000). GI domain-mediated association of the eukaryotic initiation factor 2alpha kinase GCN2 with its activator GCN1 is required for general amino acid control in budding yeast. J. Biol. Chem..

[15] Marton M.J., Vazquez de Aldana C.R., Qiu H., Chakraburtty K., Hinnebusch A.G. (1997). Evidence that GCN1 and GCN20, translational regulators of GCN4, function on elongating ribosomes in activation of eIF2alpha kinase GCN2. Mol. Cell Biol..

[16] Pochopien A.A., Beckert B., Kasvandik S., Berninghausen O., Beckmann R., Tenson T. (2021). Structure of Gcn1 bound to stalled and colliding 80S ribosomes. Proc. Natl. Acad. Sci. U. S. A..

[17] Anthony T.G., McDaniel B.J., Byerley R.L., McGrath B.C., Cavener D.R., McNurlan M.A. (2004). Preservation of liver protein synthesis during dietary leucine deprivation occurs at the expense of skeletal muscle mass in mice deleted for eIF2 kinase GCN2. J. Biol. Chem..

[18] Emanuelli G., Nassehzadeh-Tabriz N., Morrell N.W., Marciniak S.J. (2020). The integrated stress response in pulmonary disease. Eur. Respir. Rev..

[19] Abou Hassan O.K., Haidar W., Arabi M., Skouri H., Bitar F., Nemer G. (2019). Novel EIF2AK4 mutations in histologically proven pulmonary capillary hemangiomatosis and hereditary pulmonary arterial hypertension. BMC Med. Genet..

[20] Best D.H., Sumner K.L., Austin E.D., Chung W.K., Brown L.M., Borczuk A.C. (2014). EIF2AK4 mutations in pulmonary capillary hemangiomatosis. Chest.

[21] Eyries M., Montani D., Girerd B., Perret C., Leroy A., Lonjou C. (2014). EIF2AK4 mutations cause pulmonary veno-occlusive disease, a recessive form of pulmonary hypertension. Nat. Genet..

[22] Mendonsa S., von Kuegelgen N., Bujanic L., Chekulaeva M. (2021). Charcot-Marie-Tooth mutation in glycyl-tRNA synthetase stalls ribosomes in a pre-accommodation state and activates integrated stress response. Nucl. Acids Res..

[23] Spaulding E.L., Hines T.J., Bais P., Tadenev A.L.D., Schneider R., Jewett D. (2021). The integrated stress response contributes to tRNA synthetase-associated peripheral neuropathy. Science.

[24] Cordova R.A., Misra J., Amin P.H., Klunk A.J., Damayanti N.P., Carlson K.R. (2022). GCN2 eIF2 kinase promotes prostate cancer by maintaining amino acid homeostasis. Elife.

[25] Robert F., Williams C., Yan Y., Donohue E., Cencic R., Burley S.K. (2009). Blocking UV-induced eIF2alpha phosphorylation with small molecule inhibitors of GCN2. Chem. Biol. Drug Des..

[26] Nakamura A., Nambu T., Ebara S., Hasegawa Y., Toyoshima K., Tsuchiya Y. (2018). Inhibition of GCN2 sensitizes ASNS-low cancer cells to asparaginase by disrupting the amino acid response. Proc. Natl. Acad. Sci. U. S. A..

[27] Fujimoto J., Kurasawa O., Takagi T., Liu X., Banno H., Kojima T. (2019). Identification of novel, potent, and orally available GCN2 inhibitors with type I half binding mode. ACS Med. Chem. Lett..

[28] Roskoski R. (2016). Classification of small molecule protein kinase inhibitors based upon the structures of their drug-enzyme complexes. Pharmacol. Res..

[29] Tang C.P., Clark O., Ferrarone J.R., Campos C., Lalani A.S., Chodera J.D. (2022). GCN2 kinase activation by ATP-competitive kinase inhibitors. Nat. Chem. Biol..

[30] Poulikakos P.I., Zhang C., Bollag G., Shokat K.M., Rosen N. (2010). RAF inhibitors transactivate RAF dimers and ERK signalling in cells with wild-type BRAF. Nature.

[31] Keller T.L., Zocco D., Sundrud M.S., Hendrick M., Edenius M., Yum J. (2012). Halofuginone and other febrifugine derivatives inhibit prolyl-tRNA synthetase. Nat. Chem. Biol..

[32] Misra J., Holmes M.J., E T.M., Langevin M., Kim H.G., Carlson K.R. (2021). Discordant regulation of eIF2 kinase GCN2 and mTORC1 during nutrient stress. Nucl. Acids Res..

[33] Vattem K.M., Wek R.C. (2004). Reinitiation involving upstream ORFs regulates ATF4 mRNA translation in mammalian cells. Proc. Natl. Acad. Sci. U. S. A..

[34] Novoa I., Zeng H., Harding H.P., Ron D. (2001). Feedback inhibition of the unfolded protein response by GADD34-mediated dephosphorylation of eIF2alpha. J. Cell Biol..

[35] Sidrauski C., Acosta-Alvear D., Khoutorsky A., Vedantham P., Hearn B.R., Li H. (2013). Pharmacological brake-release of mRNA translation enhances cognitive memory. Elife.

[36] Wong Y.L., LeBon L., Basso A.M., Kohlhaas K.L., Nikkel A.L., Robb H.M. (2019). eIF2B activator prevents neurological defects caused by a chronic integrated stress response. eLife.

[37] Tsai Jordan C., Miller-Vedam Lakshmi E., Anand Aditya A., Jaishankar P., Nguyen Henry C., Renslo Adam R. (2018). Structure of the nucleotide exchange factor eIF2B reveals mechanism of memory-enhancing molecule. Science.

[38] Krishnamoorthy T., Pavitt Graham D., Zhang F., Dever Thomas E., Hinnebusch Alan G. (2001). Tight binding of the phosphorylated α subunit of initiation factor 2 (eIF2α) to the regulatory subunits of guanine nucleotide exchange factor eIF2B is required for inhibition of translation initiation. Mol. Cell Biol..

[39] Zyryanova Alisa F., Weis F., Faille A., Alard Akeel A., Crespillo-Casado A., Sekine Y. (2018). Binding of ISRIB reveals a regulatory site in the nucleotide exchange factor eIF2B. Science.

[40] Jiang H.Y., Wek S.A., McGrath B.C., Lu D., Hai T., Harding H.P. (2004). Activating transcription factor 3 is integral to the eukaryotic initiation factor 2 kinase stress response. Mol. Cell Biol..

[41] Zhang P., McGrath B.C., Reinert J., Olsen D.S., Lei L., Gill S. (2002). The GCN2 eIF2alpha kinase is required for adaptation to amino acid deprivation in mice. Mol. Cell Biol..

[42] Zhu S., Sobolev A.Y., Wek R.C. (1996). Histidyl-tRNA synthetase-related sequences in GCN2 protein kinase regulate *in vitro* phosphorylation of eIF-2. J. Biol. Chem..

[43] Nameki N., Yoneyama M., Koshiba S., Tochio N., Inoue M., Seki E. (2004). Solution structure of the RWD domain of the mouse GCN2 protein. Protein Sci..

[44] Sood R., Porter A.C., Olsen D.A., Cavener D.R., Wek R.C. (2000). A mammalian homologue of GCN2 protein kinase important for translational control by phosphorylation of eukaryotic initiation factor-2alpha. Genetics.

[45] Romano P.R., Garcia-Barrio M.T., Zhang X., Wang Q., Taylor D.R., Zhang F. (1998). Autophosphorylation in the activation loop is required for full kinase activity *in vivo* of human and yeast eukaryotic initiation factor 2alpha kinases PKR and GCN2. Mol. Cell Biol..

[46] Ramirez M., Wek R.C., Aldana C.R. V.d., Jackson B.M., Freeman B., Hinnebusch A.G. (1992). Mutations activating the yeast eIF-2 alpha kinase GCN2: Isolation of alleles altering the domain related to histidyl-tRNA synthetases. Mol. Cell Biol..

[47] Li B.B., Qian C., Gameiro P.A., Liu C.-C., Jiang T., Roberts T.M. (2018). Targeted profiling of RNA translation reveals mTOR-4EBP1/2-independent translation regulation of mRNAs encoding ribosomal proteins. Proc. Natl. Acad. Sci. U. S. A..

[48] Paetz W., Nass G. (1973). Biochemical and immunological characterization of threonyl-tRNA synthetase of two borrelidin-resistant mutants of Escherichia coli K12. Eur. J. Biochem..

[49] Gerken S.C., Arfin S.M. (1984). Chinese hamster ovary cells resistant to borrelidin overproduce threonyl-tRNA synthetase. J. Biol. Chem..

[50] Sattlegger E., Hinnebusch A.G. (2000). Separate domains in GCN1 for binding protein kinase GCN2 and ribosomes are required for GCN2 activation in amino acid-starved cells. EMBO J..

[51] Kato Y., Kunimasa K., Takahashi M., Harada A., Nagasawa I., Osawa M. (2020). GZD824 inhibits GCN2 and sensitizes cancer cells to amino acid starvation stress. Mol. Pharmacol..

[52] Wang R., Zheng X., Zolfaghari P., Puca L., Klippel-Giese A., Kesicki E.A. (2021).

[53] Garriz A., Qiu H., Dey M., Seo E.J., Dever T.E., Hinnebusch A.G. (2009). A network of hydrophobic residues impeding helix alphaC rotation maintains latency of kinase Gcn2, which phosphorylates the alpha subunit of translation initiation factor 2. Mol. Cell Biol..

[54] Dey M., Cao C., Dar A.C., Tamura T., Ozato K., Sicheri F. (2005). Mechanistic link between PKR dimerization, autophosphorylation, and eIF2α substrate recognition. Cell.

[55] Lavoie H., Thevakumaran N., Gavory G., Li J.J., Padeganeh A., Guiral S. (2013). Inhibitors that stabilize a closed RAF kinase domain conformation induce dimerization. Nat. Chem. Biol..

[56] Peng W., Robertson L., Gallinetti J., Mejia P., Vose S., Charlip A. (2012). Surgical stress resistance induced by single amino acid deprivation requires Gcn2 in mice. Sci. Transl Med..

