# Supplementary material for: Activation of Gcn2 by small molecules designed to be inhibitors
Source: J Biol Chem. 2023 Mar 8;299(4):104595. doi: 10.1016/j.jbc.2023.104595 (PMC10124904; doi:10.1016/j.jbc.2023.104595)
Supplement: Supporting information [file mmc1.docx]

**SUPPORTING INFORMATION**

Activation of Gcn2 by small molecules designed to be inhibitors

Kenneth R. Carlson^1^, Millie M. Georgiadis^1,2^, Feven Tameire3, Kirk A. Staschke^1,2^, and Ronald C. Wek^1,2^

^1^. Department of Biochemistry and Molecular Biology, Indiana University School of Medicine, Indianapolis, IN 46202, USA.

^2^. Indiana University Melvin and Bren Simon Comprehensive Cancer Center, Indianapolis, IN 46202, USA.

^3^. HiberCell, Inc., 619 West 54th Street, New York, NY 10019, USA

**Supplemental Figure 1:**  Overexpression of Gadd34 blocks *Atf4* translational expression induced by Gcn2iB. HEK293 cells were transfected with Atf4-Luc plasmids and nano-luciferase for normalization, along with co-expression of a Gadd34 expressing plasmid or vector alone. Transfected cells were treated with 2 µM Gcn2iB or vehicle (DMSO) for 3 or 6 hours. Atf4-Luc activities were determined and are presented in a bar graph normalized to vehicle. Error bars represent the standard error of n=4 biological replicates. The * symbols indicate a statistically significant change in luciferase activity as determined by two-way ANOVA and Tukey’s multiple comparisons test, with a single pooled variance, where ns = not significant, p > 0.05; *, p ≤ 0.05; ****, p ≤ 0.0001.
